# The long-chain flavodoxin FldX1 improves the biodegradation of 4-hydroxyphenylacetate and 3-hydroxyphenylacetate and counteracts the oxidative stress associated to aromatic catabolism in *Paraburkholderia xenovorans*

**DOI:** 10.1186/s40659-024-00491-4

**Published:** 2024-04-01

**Authors:** Laura Rodríguez-Castro, Roberto E. Durán, Valentina Méndez, Flavia Dorochesi, Daniela Zühlke, Katharina Riedel, Michael Seeger

**Affiliations:** 1 Laboratorio de Microbiología Molecular y Biotecnología Ambiental, Departamento de Química & Centro de Biotecnología Daniel Alkalay Lowitt, Universidad Técnica Federico Santa María, Avenida España 1680, 2390123 Valparaíso, Chile; 2 Millenium Nucleus Bioproducts, Genomics and Environmental Genomics (BioGEM), Avenida España 1680, 2390123 Valparaíso, Chile; 3 Institute of Microbiology, University of Greifswald, Felix-Hausdorff-Strasse 8, 17489 Greifswald, Germany

**Keywords:** Flavodoxin, Long-chain flavodoxin, *Paraburkholderia xenovorans* LB400, Oxidative stress, Hydroxyphenylacetate, Proteome

## Abstract

**Background:**

Bacterial aromatic degradation may cause oxidative stress. The long-chain flavodoxin FldX1 of *Paraburkholderia xenovorans* LB400 counteracts reactive oxygen species (ROS). The aim of this study was to evaluate the protective role of FldX1 in *P. xenovorans* LB400 during the degradation of 4-hydroxyphenylacetate (4-HPA) and 3-hydroxyphenylacetate (3-HPA).

**Methods:**

The functionality of FldX1 was evaluated in *P. xenovorans* p2-*fldX1* that overexpresses FldX1. The effects of FldX1 on *P. xenovorans* were studied measuring growth on hydroxyphenylacetates, degradation of 4-HPA and 3-HPA, and ROS formation. The effects of hydroxyphenylacetates (HPAs) on the proteome (LC–MS/MS) and gene expression (qRT-PCR) were quantified. Bioaugmentation with strain p2-*fldX1* of 4-HPA-polluted soil was assessed, measuring aromatic degradation (HPLC), 4-HPA-degrading bacteria, and plasmid stability.

**Results:**

The exposure of *P. xenovorans* to 4-HPA increased the formation of ROS compared to 3-HPA or glucose. *P. xenovorans* p2-*fldX1* showed an increased growth on 4-HPA and 3-HPA compared to the control strain WT-p2. Strain p2-*fldX1* degraded faster 4-HPA and 3-HPA than strain WT-p2. Both WT-p2 and p2-*fldX1* cells grown on 4-HPA displayed more changes in the proteome than cells grown on 3-HPA in comparison to glucose-grown cells. Several enzymes involved in ROS detoxification, including AhpC2, AhpF, AhpD3, KatA, Bcp, CpoF1, Prx1 and Prx2, were upregulated by hydroxyphenylacetates. Downregulation of organic hydroperoxide resistance (Ohr) and DpsA proteins was observed. A downregulation of the genes encoding scavenging enzymes (*katE* and *sodB*), and *gstA* and *trxB* was observed in p2-*fldX1* cells, suggesting that FldX1 prevents the antioxidant response. More than 20 membrane proteins, including porins and transporters, showed changes in expression during the growth of both strains on hydroxyphenylacetates. An increased 4-HPA degradation by recombinant strain p2-*fldX1* in soil microcosms was observed*.* In soil, the strain overexpressing the flavodoxin FldX1 showed a lower plasmid loss, compared to WT-p2 strain, suggesting that FldX1 contributes to bacterial fitness. Overall, these results suggest that recombinant strain p2-*fldX1* is an attractive bacterium for its application in bioremediation processes of aromatic compounds.

**Conclusions:**

The long-chain flavodoxin FldX1 improved the capability of *P. xenovorans* to degrade 4-HPA in liquid culture and soil microcosms by protecting cells against the degradation-associated oxidative stress.

**Supplementary Information:**

The online version contains supplementary material available at 10.1186/s40659-024-00491-4.

## Introduction

Bacteria that inhabit aerobic environments are exposed to the harmful effects of reactive oxygen species (ROS), such as hydrogen peroxide (H_2_O_2_), superoxide radical (O_2_^−^), and hydroxyl radical (OH˙), which are intracellularly generated by the reduction of molecular oxygen [1–3]. ROS may generate cellular damage disrupting iron-cofactor enzymes, and oxidizing DNA, proteins, and lipids [3, 4]. Regulation of membrane permeability, antioxidant and repair systems, ROS-scavenging enzymes, and replacement of ROS-sensitive targets by resistant isofunctional enzymes are part of the adaptive mechanisms to tolerate aerobic environments, capable of counteracting the internal oxidation to nontoxic levels [3, 4]. Interestingly, H_2_O_2_ is also an anaerobic electron acceptor for *E. coli* in the absence of molecular oxygen by the respiratory oxidase cytochrome c peroxidase, reducing its toxicity [5]. Electron-active metabolites such as phenazines, which are produced under nutrient limitation in *Pseudomonas*, could serve as electron acceptors for the reoxidation of NADH, allowing bacteria to maintain redox homeostasis and generate energy for growth and maintenance [6].

ROS accumulation can occur in the cell when ROS levels exceed the defense mechanisms, entering to a status known as oxidative stress, inducing scavenging enzymes and other general stress proteins [2, 7]. These enzymatic systems are induced in bacteria directly by exposure to oxidizing agents, such as H_2_O_2_, superoxide or NO, and indirectly via a general stress response caused by environmental factors and chemical agents [4, 8–11]. Exposure to environmental stresses can cause a general detriment in cellular growth, performance, survival and fitness, partly related to ROS accumulation and oxidative damage. Similarly, aerobic catabolism and exposure to aromatic compounds of *Paraburkholderia*, *Pseudomonas*, *Bacillus*, and *Acinetobacter* strains showed increased general stress and oxidative stress proteins [8, 10, 12–16]. On the other hand, aromatic catabolic pathways often involve redox modifications on the substrate, such as mono- or dioxygenations performed by Rieske nonheme iron oxygenases [17]. Due to their mechanism of action, when novel aromatic substrates do not fit properly in the active enzyme center of mono- or dioxygenases, ROS are released [2, 18, 19].

*Paraburkholderia xenovorans* LB400 is a model bacterium for the degradation of polychlorobiphenyls (PCBs) and diverse aromatic compounds [20–27]. The LB400 genome contains genes encoding for oxygenases, dioxygenases, and hydroxylases, key enzymes involved in the degradation of aromatic compounds, as well as a wide repertoire of genes involved in oxidative stress response [4, 24]. Exposure of *P. xenovorans* LB400 to (chloro)biphenyls (*i.e.*, biphenyl, 4-chlorobiphenyl), chlorobenzoates (*i.e.*, 2-chlorobenzoate, 4-chlorobenzoate) and *p*-cymene increases the expression of molecular chaperones (DnaK, GroEL, HtpG, ClpB), the alkyl hydroperoxide reductase (AhpC), the organic hydroperoxide resistance protein (Ohr), membrane proteins and enzymes involved in energy production processes, indicating that the catabolism of aromatic compounds induces an oxidative stress response [8, 10, 14].

Induction of electron shuttles, as flavodoxins, is a common feature of the antioxidant response for cell redox balance. Flavodoxins are small electronic transfer flavoproteins, highly isofunctional to ferredoxins, expressed under conditions of oxidative stress and iron limitation [7, 28, 29]. Ferredoxins possess an iron-sulfur cluster, sensitive to ROS, while flavodoxins have a flavin mononucleotide as a prosthetic group, which possesses higher resistance to ROS. Flavodoxins possess a distinctive secondary structure, consisting of five ß-sheet intercalated with five α-helices [30]. Previously, we described two flavodoxins in strain LB400, FldX1 and FldX2 [29]. Overexpression of the long-chain flavodoxin FldX1 of *P. xenovorans* LB400 improved its resistance to the redox-cycling compound paraquat and H_2_O_2_, showing a more relevant antioxidant role than the short-chain flavodoxin FldX2 [29]. For this reason, the protective role of FldX1 during aromatic compounds (4-HPA and 3-HPA) degradation in strain LB400 was assessed. In this study, degradation pathways in *P. xenovorans* were studied. Catabolism of 4-HPA is environmentally important because it is a product of lignin decomposition and an industrial pollutant present in wastewater from olive oil production [31].

The aim of this study was to evaluate the protective role of the long-chain flavodoxin FldX1 during the catabolism of 4-HPA and 3-HPA in *P. xenovorans* strain LB400. The growth of *P. xenovorans* overexpressing FldX1 and its catabolism of HPAs in liquid culture was studied. In addition, the ROS formation, the proteome and gene expression were analyzed during the degradation of 4-HPA and 3-HPA. Furthermore, the bioremediation potential of the strain overexpressing FldX1 was evaluated in soil microcosms polluted with 4-HPA.

## Methods

### Chemicals

The aromatic compounds 4-HPA and 3-HPA were obtained from Sigma-Aldrich (purity 98%; St. Louis, MO, United States).

### Bacterial strains and culture conditions

In a previous study of our group [29], a recombinant strain for the overexpression of FldX1 was constructed. Therefore, the gene encoding the flavodoxin FldX1 (BxeA0278) of *P. xenovorans* LB400 was cloned in the vector pBBR1MCS-2. The resulting plasmid called p2-fldX1 was transformed in *E. coli* S17λpir and transferred from this strain to *P. xenovorans* LB400 by mating. The resulting strain was called *p2-fldX1*. A control strain (WT-p2), carrying the plasmid pBBR1MCS-2 was also constructed. qRT-PCR revealed that the *fldX1* gene expression was highly induced (> 600-fold) in recombinant strain p2-fldX1 in comparison to the control strain [29].

*P. xenovorans* overexpressing the flavodoxin FldX1 (strain p2-*fldX1*) and the control strain WT-p2 (carrying the empty vector) [29] were grown at 30 °C in Luria–Bertani (LB) modified medium (5 g L^−1^ tryptone, 2.5 g L^−1^ yeast extract and 2.5 g L^−1^ NaCl) or in M9 minimal medium with glucose, 3-HPA or 4-HPA (5 mM) as sole carbon and energy source, supplemented with kanamycin (25 μg mL^−1^). Growth was determined by measuring turbidity at 600 nm or by counting colony-forming units mL^−1^ (CFU mL^−1^).

### Bacterial growth assays

Recombinant strains were grown overnight in modified LB medium at 30 °C. Cells were washed with 0.9% NaCl and used as inoculum for M9 minimal medium with glucose, 3-HPA or 4-HPA (5 mM) as sole carbon and energy sources. Recombinant strains were cultivated at 30 °C and growth was determined by measuring turbidity at 600 nm. Bacterial growth data were analyzed by independent one-tailed Student's t-test (p ≤ 0.05). All analyses were performed using R version 4.3.3.

### Aromatic compounds degradation assays

Recombinant strains were cultivated for 24 h at 30 °C in M9 minimal medium with 3-HPA or 4-HPA (5 mM) as sole carbon and energy sources. Samples were taken every 3 h to quantify the aromatic compounds by high-performance liquid chromatography coupled to a diode array detector (HPLC–DAD), using a C-18 column. The mobile phase consisted of methanol:formic acid 0.1% (30:70) with a flux of 1 mL min^−1^. The compounds 3-HPA and 4-HPA were measured at 272 and 270 nm, respectively. The retention times of 3-HPA and 4-HPA were 3.5 and 3.1 min, respectively. HPAs degradation data were analyzed by a one-way analysis of variance (ANOVA). The means were compared by Tuckey’s HSD test (p ≤ 0.05) using R version 4.3.3. No analysis was performed when a group mean was equal to 0, as normality of data is affected.

### Reactive oxygen species formation

Quantification of the hydroxyl (OH˙) radical was carried out in a similar way to that described by Cárdenas [32], using the fluorescent probe 3'-(p-hydroxyphenyl) fluorescein (HPF) (Life Technologies, USA) specific for the OH ˙ radical detection. For the determination of OH ˙ generated by aromatic compounds, *P. xenovorans* was grown at 30ºC until reaching the exponential phase (turbidity at 600 nm ~ 0.4) in M9 minimal medium supplemented with glucose (5 mM) as the only source of carbon and energy. Bacteria were incubated for 1 h in a ratio of 1:1000 with the HPF probe, and subsequently, cells were incubated at 30ºC in the presence of glucose, 3-HPA or 4-HPA (5 mM), where the fluorescence of the HPF probe was measured at an emission wavelength (ʎ_emission_) of 515 nm and an excitation wavelength (ʎ_excitation_) of 490 nm.

### Protein expression analysis

*P. xenovorans* recombinant strains (p2-*fldX1* and WT-p2) were grown in M9 minimal medium with glucose, 3-HPA or 4-HPA (5 mM) as sole carbon and energy sources until exponential phase. Bacterial cells were harvested and washed with 1 mL of TE buffer (pH 7.5). Cells were suspended in 1 mL TE buffer and subjected to mechanical disruption with 500 µL of glass beads (0.1–0.11 mm, Sartorius Stedim Biotech, Göttingen, Germany) in a FastPrep-24 homogenizer (MP Biomedicals, Santa Ana, USA) in three cycles (3 × 30 s at 6.5 m/s). Samples were centrifuged for 10 min (21,500 *g* at 4 °C), and the supernatant was lyophilized for further analysis. This experiment included six replicates per condition.

Protein pellets were resolved in 100 µl 8 M urea/2 M thiourea and the concentration was determined using Roti Nanoquant (Carl Roth, Karlsruhe, Germany). Protein extracts were digested using trypsin via suspension trapping (S-trap micro columns; ProtFi, Huntington, NY, USA) according to the manufacturer’s instructions. Briefly, 50 µg of protein extract were filled up with SDS lysis buffer to achieve a final concentration of 5% SDS. Subsequently, proteins were reduced with 10 mM dithiothreitol (DTT, Sigma Aldrich, St. Louis, USA), alkylated with 20 mM iodoacetamide (IAA, Sigma Aldrich, St. Louis, USA) and acidified using phosphoric acid (Carl Roth, Karlsruhe, Germany). Samples were diluted with S-Trap buffer (90% methanol, 100 mM triethylammonium bicarbonate (TEAB, Sigma Aldrich, St. Louis, USA) in a 1:7 ratio before loading onto S-trap columns. After loading, protein samples were washed four times and activated trypsin (Promega, Madison, USA) was added in a trypsin:protein ratio of 1:50. After tryptic digestion for 3 h at 47 °C peptides were eluted using 50 mM TEAB (elution step 1), 0.1% acetic acid (elution step 2) and 60% acetonitrile/0.1% acetic acid (elution step 3). Eluted peptides were pooled and dried in a vacuum centrifuge. For purification, peptides were resolved in 300 µl 0.1% TFA and loaded onto self-packed C18 spin columns as described [33]. After washing the peptides were eluted by an increasing acetonitrile gradient, yielding eight fractions. Fractions 1 and 5, 2 and 6, 3 and 7, 4 and 8 were pooled, dried in a vacuum centrifuge and finally resolved prior to mass spectrometry analysis in 20 µl 0.1% acetic acid.

LC–MS/MS analyses were done using an EASY-nLC 1200 coupled to a Q Exactive HF Orbitrap mass spectrometer (Thermo Fisher Scientific, Waltham, USA). Peptides were loaded directly onto a self-packed analytical column with an integrated emitter (20 cm, 0.1 mm diameter, packed with 3 µm C18 (Dr. Maisch, Ammerbuch-Entringen, Germany) using buffer A (0.1% acetic acid). Separation of peptides was achieved using a binary gradient from 5 to 50% buffer B (0.1% acetic acid in acetonitrile) and a constant flow rate of 300 nL/min. Survey scans were recorded in the Orbitrap with a resolution of 60.000 from 333 to 1650 m/z. The 15 most intense peaks per scan cycle were subjected to fragmentation. Dynamic exclusion of precursor ions was enabled for 30 s; single-charged ions and ions with unknown charge state were excluded from fragmentation. Internal lock-mass calibration was applied (445.12003).

Protein identification and label-free quantification (LFQ) were done using the MaxQuant proteomics software package v1.6.17.0 [34] and a protein sequence database containing all protein sequences of *P. xenovorans* LB400 (8317 entries, NCBI, v2021-12-08). Reverse sequences and common contaminants were added by the software. Parameters were set as follows: Trypsin cleavage with a maximum of two missed cleavages, fixed modification Carbamidomethyl (C), variable modification Oxidation (M), and default parameters were used for protein identification and label-free quantification. For evaluation and statistical analysis of the data the MaxQuant companion software Perseus was used [35]. Proteins were considered if they were present in four out of six replicates. Averaged LFQ values were used to calculate fold changes. Proteins with significantly different amounts between the treatments were identified by t-tests (FDR 0.01). Visualization of proteome data using Voronoi tree maps was done using Paver software (Decodon, Greifswald, Germany). The mass spectrometry proteomics data have been deposited to the ProteomeXchange Consortium via the PRIDE partner repository, with the dataset identifier PXD047097 [36].

### Gene expression analysis

Differential gene expression was analyzed by qRT-PCR. *P. xenovorans* recombinant strains were grown in M9 minimal medium with 3-HPA or 4-HPA (5 mM) as sole carbon and energy source until exponential phase. Bacterial cells were harvested and the RNA was extracted using the RNeasy Mini Kit (Qiagen, Hilden, Germany), following the manufacturer’s instructions. Residual DNA was removed employing the TURBO DNA-free Kit (Thermo Fisher Scientific; Waltham, MA, USA). RNA quality was analyzed with the NanoDrop One spectrometer (Thermo Fisher Scientific; Waltham, MA, USA). RNA integrity was checked in a 1% w v^−1^ agarose gel. cDNA was synthetized with the First Strand cDNA Synthesis Kit (Thermo Fisher Scientific; Waltham, MA, USA). The qRT-PCRs were carried out with the KAPA SYBR FAST qPCR Master Mix Kit (Kapa Biosystems; Boston, MA, USA), following the manufacturer’s instructions. The *gyrB* and *ftsZ* genes were used as reference genes. Quantitative RT-PCR analysis was performed on a Mx3000P qPCR system (Stratagene, Agilent Technologies, Santa Clara, California, USA). The results were analyzed using the Hellemans’ method [37]. The primers used in this study are listed in Additional file 1: Table S1.

### Soil microcosms studies

#### Experiment set-up

Soil with no pollution history was collected from Quilpué, Valparaíso Region, Chile (− 33.052278, − 71.411154). Soil was sifted using 4.75- and 2-mm pore diameter sieves to remove small rocks, plant parts, and small organism rests. Five treatments were included in this assay (Table 1), with three replicates in each case. Glass jars containing 80 g of soil were autoclaved twice to decrease the concentration of soil native microbiota. For those treatments including 4-HPA, the compound was added to the soil at a final concentration of 10 mM. Table 1 shows the experimental set-up that included five conditions. For the determination of total heterotrophs, bacterial cells were extracted from soil and plated in modified LB medium.

**Table 1:** **Experimental design for soil microcosm experiments**

Microcosm code	Treatment	4-HPA	Strain
		(10 mM)	
T1	Uninoculated	+	–
T2	Bioaugmented	+	*P. xenovorans* p2-*fldX1*
T3	Bioaugmented	+	*P. xenovorans* WT-p2
T4	Bioaugmented	–	*P. xenovorans* p2-*fldX1*
T5	Bioaugmented	–	*P. xenovorans* WT-p2

To inoculate the microcosms, *P. xenovorans* recombinant strains were grown until the exponential phase in M9 minimal medium on pyruvate (10 mM) as sole carbon and energy source at 30 °C. Cells were washed with 0.9% NaCl and inoculated in the soil at a concentration of 1.5 × 10^7^ cells/g of dry soil, in the corresponding microcosms.

#### Quantification of bacteria and 4-HPA determination

The assay was performed at room temperature for 3 weeks. Every 7 days, samples were taken to quantify heterotroph bacteria and 4-HPA-degrading cells. Soil humidity was determined, and maintained at 30–35%, adding sterile water when needed. Every 3 or 4 days, samples were taken to analyze 4-HPA degradation.

To quantify bacteria, 1 g of soil was mixed with 9 mL of 0.9% NaCl and vigorously shaken for 1 h. Samples were taken to perform 1/10-serial dilutions. To determine total heterotrophs, cells were plated in LB modified medium. For the determination of 4-HPA-degrading bacteria and plasmid prevalence, cells were plated in M9 minimal medium with 4-HPA (5 mM) as sole carbon and energy sources, supplemented with or without kanamycin (25 μg mL^−1^). Plates were incubated at room temperature for 48–72 h and CFU g^−1^ of dry soil were determined.

To quantify 4-HPA degradation, 1 g of soil was mixed with 1 g of (NH_4_)_2_SO_4_ and 5 mL de 30% methanol. The mixes were vigorously shaken for 1 h and sonicated for 15 min. The samples were centrifuged for 20 min at 3200 *g* at 4 °C to sediment soil particles and (NH_4_)_2_SO_4_. Supernatants were filtered with a 0.22 µm-pore filter to remove bacterial cells. Remaining 4-HPA was quantified using an HPLC–DAD with a C-18 column. The mobile phase used was methanol:formic acid 0.1% (18:82) with a flux of 1 mL min^−1^. 4-HPA was measured at 270 nm and showed a retention time of 5.3 min.

## Results

### Exposure upon 4-HPA increases ROS formation in comparison to 3-HPA

**Fig. 1:**
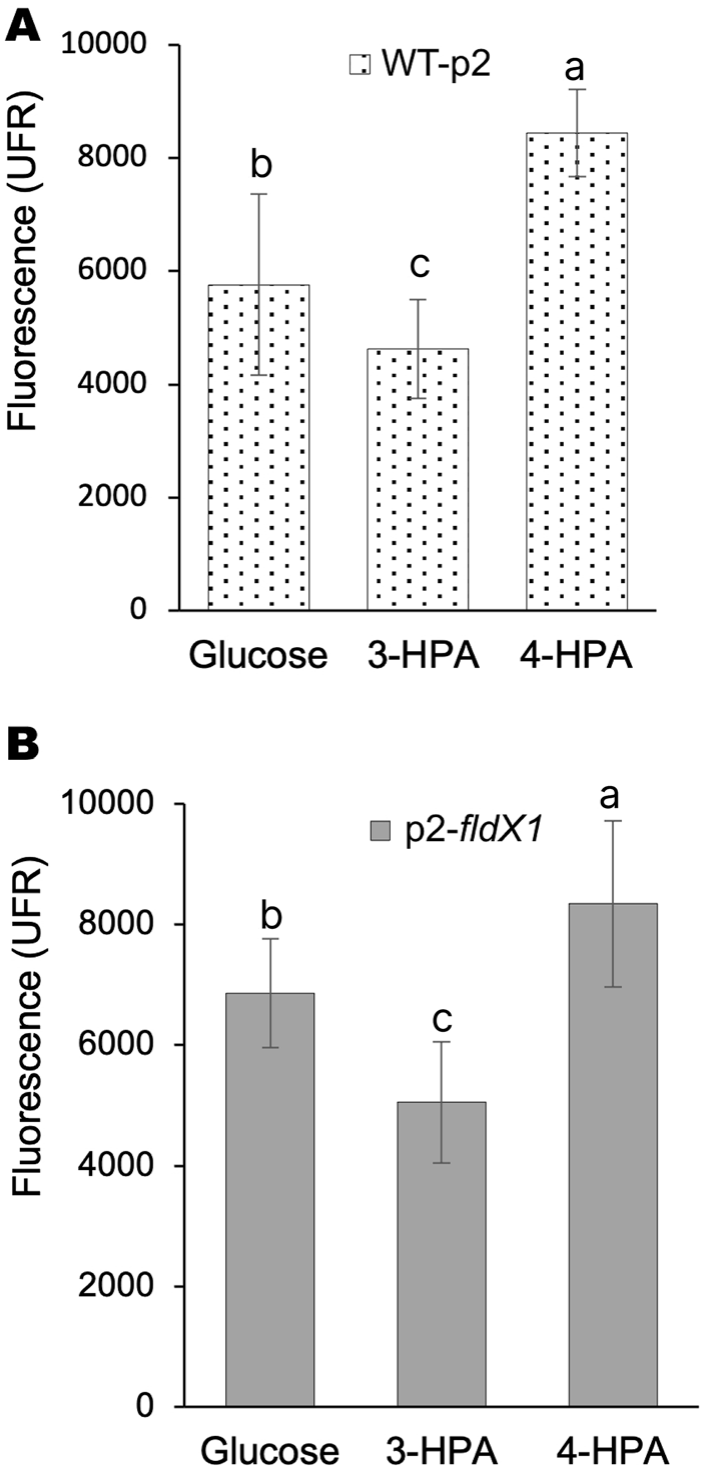
ROS formation in *P. xenovorans* recombinant strains upon 4-HPA and 3-HPA exposure. Cells were grown on glucose until exponential phase and incubated with 3-HPA or 4-HPA (5 mM). **A,** strain WT-p2. **B,** strain p2-*fldX1*. Unexposed cells were also measured (control). Each value is an average ± SD of at least three independent experiments. Significant differences between groups were evaluated using one-way ANOVA followed by a Fischer's least significant difference (LSD) test (α = 0.05)

To determine how the aromatic compounds HPAs, specifically 4-HPA and 3-HPA, contribute to oxidative stress, ROS formation of *P. xenovorans* grown on these compounds was analyzed. Figure 1 shows that incubation of both WT-p2 and p2-*fldX1* cells with 4-HPA generates more hydroxyl radicals compared to 3-HPA and to glucose (control).

### The flavodoxin FldX1 improves growth and degradation of 3-HPA and 4-HPA

**Fig. 2:**
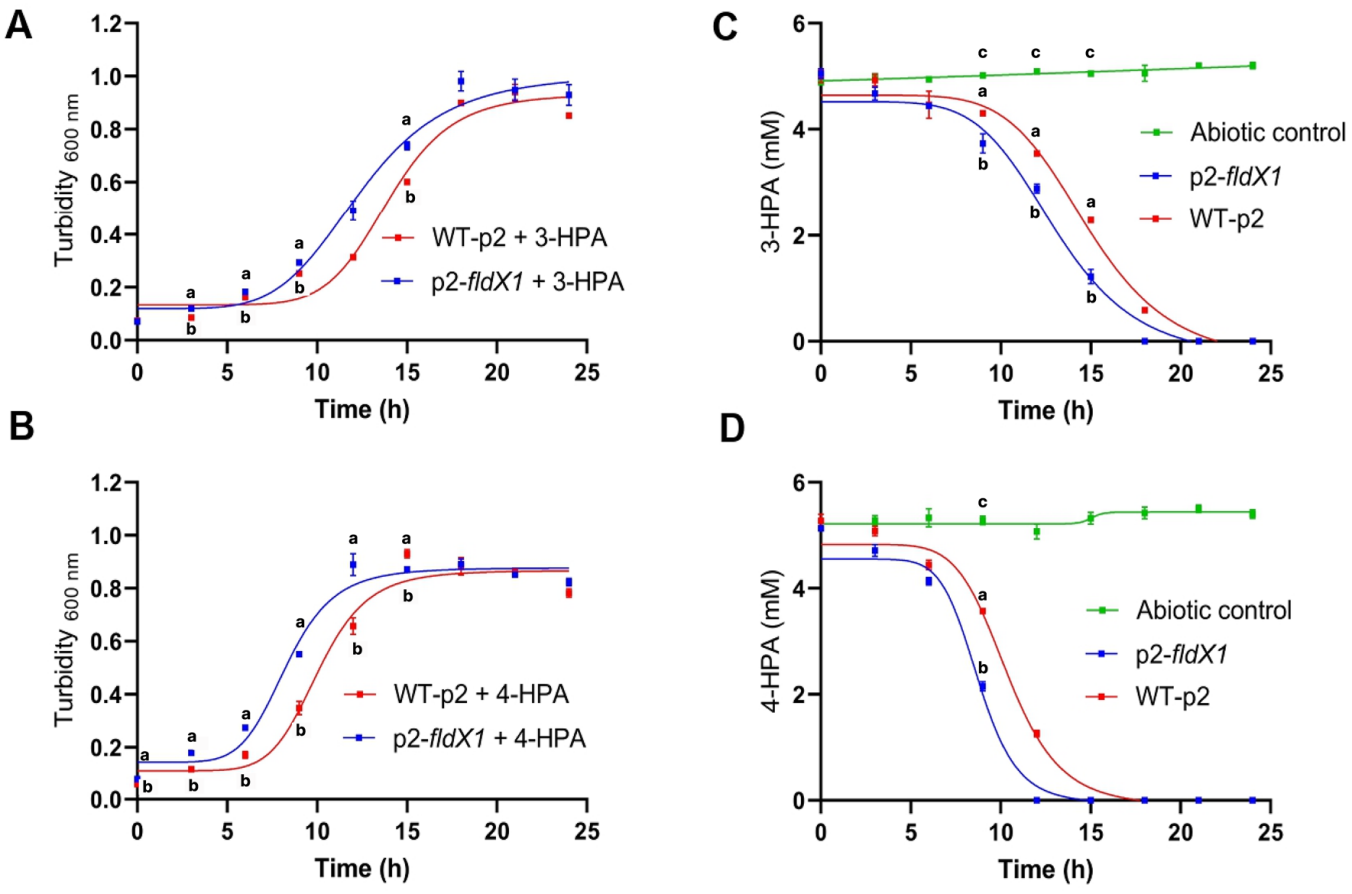
Effects of FldX1 on growth and degradation of 3-HPA and 4-HPA. Cells (p2-*fldX1* and WT-p2) were grown for 24 h in M9 minimal medium on (**A)** 3-HPA or (**B**) 4-HPA as sole carbon and energy sources. Growth was determined measuring turbidity at 600 nm. Different letters within a time point indicate significant differences from each other according to Student’s t-test, at p ≤ 0.05. 3-HPA and 4-HPA concentrations were determined in the supernatants by HPLC–DAD. (**C**) 3-HPA. **D** 4-HPA. Different letters within a time point indicate significant differences from each other according to one-way ANOVA followed by Tuckey´s HSD

To evaluate the effect of FldX1 on the growth of *P. xenovorans* on aromatic compounds, the recombinant strains were grown in M9 minimal medium using 3-HPA or 4-HPA (5 mM) as sole carbon and energy sources. During growth on 3-HPA (Fig. 2A) strain overexpressing the flavodoxin FldX1 showed a higher growth rate (0.074 h^−1^) than the control strain (0.058 h^−1^). Strain p2-*fldX1* reached the stationary phase after 18 h incubation, while strain WT-p2 reached this phase after 21 h. Similarly, during growth on 4-HPA (Fig. 2B), strain p2-*fldX1* exhibited a higher growth rate (0.119 h^−1^) than strain WT-p2 (0.081 h^−1^). Strain overexpressing the flavodoxin FldX1 reached the stationary phase after 12 h incubation, while the strain WT-p2 reached this phase at ~ 14 h. Interestingly, both strains (p2-*fldX1* and WT-p2) grew faster on 4-HPA as sole carbon and energy sources compared to 3-HPA. This result is in accordance with a previous study carried out with the wild-type strain LB400, where the culture reached a higher cell density growing on 4-HPA as sole carbon an energy source in comparison to 3-HPA [25].

To evaluate the effect of FldX1 on the degradation capabilities of p2-*fldX1* and WT-p2 strains, 3-HPA or 4-HPA were quantified in culture supernatants by HPLC–DAD. Strain p2-*fldX1*, overexpressing the flavodoxin FldX1, showed an improved 3-HPA degradation compared to the control strain (Fig. 2C). 3-HPA was completely degraded by strain p2-*fldX1* at 18 h of incubation, whereas the strain WT-p2 completely degraded 3-HPA at 21 h. Figure 2D shows that p2-*fldX1* cells also exhibited a higher 4-HPA degradation compared to the control strain. Interestingly, 4-HPA was completely degraded by the strain p2-*fldX1* earlier than 3-HPA (~ 12 h), while the control strain (WT-p2) completely degraded 4-HPA at ~ 15 h. In accordance with the growth curves, both strains p2-*fldX1* and WT-p2, degraded 4-HPA faster than 3-HPA. These results indicate that the flavodoxin FldX1 confers an advantage during bacterial growth and degradation of 3-HPA and 4-HPA compounds.

#### FldX1 prevented the upregulation of stress-related proteins

To understand the effects of FldX1 overexpression on *P. xenovorans* proteome grown on 3-HPA or 4-HPA, protein expression analyses were performed using glucose as control. In this assay, a total of 3,179 proteins from *P. xenovorans* were quantified from a total of 8,754 coding sequences of the genome. Nevertheless, only proteins identified in ≥ 4 of the 6 replicates of each condition were considered for the statistical analysis, reducing the identified proteins to 2,093 proteins that fit our inclusion criteria (Additional file 1: Table S2 and Figure S1). Additional file 1: Table S2 contains all the relevant information of the identified and quantified proteins from the mass spectrometry analysis. Besides the functional annotation and classification into clusters of orthologous groups (COG) of the proteins, the quantitative data can be assessed: Label-free quantification data (LFQ) obtained from the MaxQuant software indicate the abundance of the respective protein–this can be used to rank proteins according to their abundance in a strain/condition; averaged values of LFQ and the calculated fold changes can also be drawn from this table. Also, the result of the statistical analysis obtained by the MaxQuant companion software Perseus (t-test) is indicated for each protein. The Additional file 1: Fig. S1 visualize an upset plot showing the shared and unique proteins in the different samples.

The log2-fold change indicates the change in comparison to the glucose-grown strain. The (log2) fold change is calculated based on average LFQ values within the replicates for each strain/condition. If these quantitative values within the replicates of a strain/condition vary much, this impairs statistical significance. As a result, the difference (fold change) is not significant although the averaged values lead to high induction factors. Additional file 1: Table S2 contains a column with bar charts indicating the LFQ values of each sample and allowing easy comparison of the variances of these LFQ values.

The analysis focused on comparing each strain (p2-*fldX1* and control) grown on 3-HPA or 4-HPA versus glucose-grown cells (control). A very low induction/repression ratio might not be biologically relevant even if it is statistically significant. Proteins with significantly changed amount and a log2-fold change of 1 (or − 1 for repression; corresponding to twofold change) are highlighted in the Additional file 1: Table S2 for better visualization to account for biological relevance. Proteome analysis revealed novel information about the antioxidant stress response and the physiological response to HPA catabolism. In p2-*fldX1* cells cultured in 3-HPA, 109 and 83 proteins were upregulated and downregulated compared to glucose-grown cells, respectively. When p2-*fldX1* cells were cultured on 4-HPA, 128 and 85 proteins were upregulated and downregulated, respectively. Of all these proteins, 24 and 26 of them were only detected in p2-*fldX1* cells during growth on 3-HPA and 4-HPA (“*on*”), respectively, but not in the control condition (Additional file 1: Table S3). Similarly, 21 and 25 proteins were not detected in p2-*fldX1* cells when cultured on 3-HPA and 4-HPA, respectively, in comparison to glucose (“*off*”) (Additional file 1: Table S4).

In WT-p2 cells growing on 3-HPA, 104 and 80 proteins were upregulated and downregulated, respectively, compared to glucose-grown cells. When strain WT-p2 was cultured on 4-HPA, 171 and 125 proteins were upregulated and downregulated, respectively. Of all these proteins, 24 and 24 were “*on*” during growth on 3-HPA and 4-HPA, respectively, and 21 and 24 were “*off*” during the same conditions, compared to glucose.

#### Upregulation of the 3-HPA and 4-HPA catabolic proteins

**Fig. 3:**
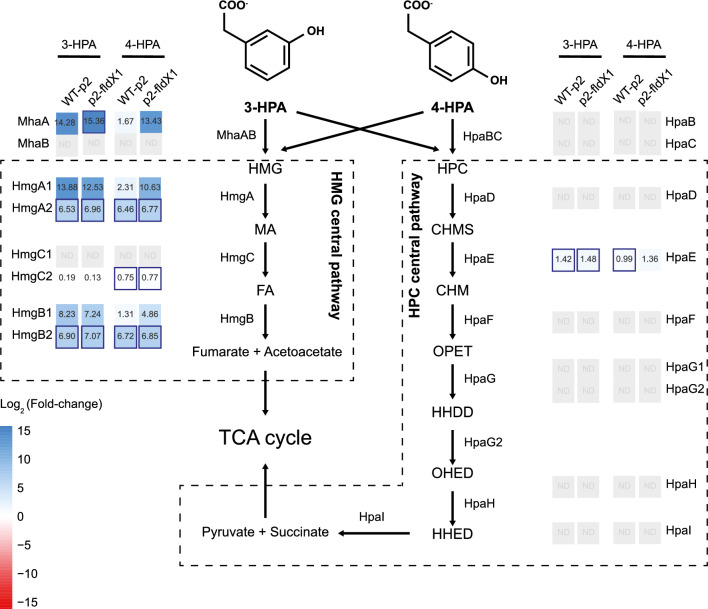
Overview of the protein expression profile for the catabolism of 3-HPA and 4-HPA in *P. xenovorans* strains. Heatmaps show changes in protein expression levels of cells grown until exponential phase on 3-HPA and 4-HPA relative to glucose as sole carbon source. Each value is an average of Log_2_(expression change) of at least four independent experiments. Significant statistically differences are represented as colored border for the corresponding strain. Peripheral and central pathways for 3-HPA and 4-HPA are depicted including their metabolic intermediates. Catabolic enzymes: MhaAB: 3-HPA 6-hydroxylase; HmgA: HMG 1,2-dioxygenase; HmgB: Fumarylacetate hydrolase; HmgC: Maleylacetate isomerase; HpaBC: 4-HPA monooxygenase; HpaD: HPC 2,3-dioxygenase; HpaE: CHMS dehydrogenase; HpaF: CHM isomerase; HpaG: OPET decarboxylase. Metabolic intermediates of 3-HPA and 4-HPA degradation: HMG: Homogentisate.; MA: Maleylacetoacetate; FA: Fumarylacetoacetate; HPC: Homoprotocatechuate; CHMS: 5-carboxymethyl-2-hydroxy-muconic semialdehyde; CHM: 5-carboxymethyl-2-hydroxy-muconic acid.; OPET: 5-oxo-pent-3-ene-1,2,5-tricarboxylic acid; HHDD: 2-hydroxy-hept-2,4-diene-1,7-dioic acid. TCA cycle: Tricarboxylic acid cycle. ND: Not determined

Of the proteins induced in p2-*fldX1* cells during HPA growth condition, aromatic catabolic proteins, and transport proteins were identified. Several enzymes of 3-HPA and 4-HPA degradation pathways were identified by proteomics. Upregulation of the 5-carboxymethyl-2-hydroxy-muconic semialdehyde (CHMS) dehydrogenase (HpaE, Bxe_B2030) protein from the homoprotocatechuate (HPC) pathway was detected on strains p2-*fldX1* and WT-p2 during growth on 3-HPA and 4-HPA (Fig. 3). HpaE transforms CHMS in 5-carboxymethyl-2-hydroxy-muconic acid (CHM). The HpaD protein (Bxe_B2031), the ring-cleavage dioxygenase of the homoprotocatechuate pathway, was not detected in the proteomic analysis. Other enzymes of this pathway were not identified or did not fit the filter criteria for quantification. In the case of the homogentisate (HMG) pathway, the subunit A (MhaA, Bxe_A2727) of the enzyme 3-HPA 6-hydroxylase (MhaAB) was upregulated in both strains during growth on 3-HPA and 4-HPA, although no significant statistically difference was calculated. Interestingly, in the case of strain WT-p2 cultured in 4-HPA, the expression of the protein was very low compared to 3-HPA. The subunit B of this (MhaB, Bxe_A2726) was not detected. The same effect was identified for the first copy of the enzyme HMG 1,2-dioxygenase (HmgA1, Bxe_A2725). The first copy of the maleylacetoacetate isomerase (HmgC2, Bxe_A4141) was significantly upregulated in both strains p2-*fldX1* and WT-p2 cultured in 4-HPA. Nevertheless, the other copy of this enzyme (HmgC1, Bxe_A2723) did not show changes in expression levels (Fig. 3). The two copies of the enzyme fumarylacetoacetate hydrolase (HmgB1 (Bxe_A2724) and HmgB2 (Bxe_A3899)) were upregulated in both strains growing on 3-HPA and 4-HPA. The induction of Hmg proteins in presence of 3-HPA and 4-HPA indicates the utilization of the homogentisate ring-cleavage pathway for the degradation of both HPAs.

Interestingly, during the growth of strains p2-*fldX1* and WT-p2 on 3-HPA and 4-HPA, almost all proteins of the phenylacetic acid degradation pathways described in *P. xenovorans* LB400 [38] were upregulated, with higher expression levels observed in 3-HPA. Catabolic proteins of the phenylacetate pathway (*paaH*, *paaI* and *paaJ*) were induced in presence of 3-HPA and 4-HPA. In addition, transcriptional analyses during growth on 3-HPA and 4-HPA, showed that during growth on 3-HPA (Additional file 1: Fig. S2), p2-*fldX1* and WT-p2 exhibited an expected increase of *hmgA1* gene transcript compared to growth on glucose (199- and 328-fold, respectively). In 3-HPA-grown cells, *hmgA2* gene increased 121- and 176-fold in the strains p2-*fldX1* and WT-p2, respectively. Interestingly, for both genes *hmgA1* and *hmgA2*, strain WT-p2 exhibited higher expression levels than strain p2-*fldX1.* This suggests that downregulation of catabolic genes in strain p2-*fldX1* may be a consequence of a minor oxidative stress condition and an improved catabolic performance, causing a decrease of intracellular 3-HPA concentration. The *hpaD* gene in both strains p2-*fldX1* and WT-p2 did not show variations in expression levels in comparison to the control, and showed low expression levels (Additional file 1: Fig. S2). This is in accordance with proteomic analyses, in which undetectable HpaD protein levels were observed probably below detection limit for MS analysis. 4-HPA catabolism showed a similar expression increase of the *hmgA1* gene expression for both strains (128 to 130-fold), compared to glucose-grown cells (Additional file 1: Fig. S2). No changes were detected in *hmgA1* and *hpaD* gene expression levels, suggesting that the higher degradation by strain p2-*fldX1* is not based on the differential expression of these ring-cleavage enzymes of the catabolic pathway.

Other proteins that probably are related to aromatic catabolism were induced in both strains (p2-*fldX1* and WT-p2) in 3-HPA and 4-HPA: an extradiol ring-cleavage dioxygenase (Bxe_A0432), an aldehyde dehydrogenase (Bxe_B2718) and an alkanesulfonate monooxygenase (Bxe_A2493) (Additional file 1: Table S3). A FAD-dependent oxidoreductase (Bxe_B1666) was upregulated in p2-*fldX1* in 3-HPA; a salicylate dehydrogenase (Bxe_C0213) was induced in p2-*fldX1* in 4-HPA and in WT-p2 in both aromatics; and a shikimate 5-dehydrogenase (Bxe_B0884) was upregulated in both strains in 4-HPA.

#### Upregulation of antioxidant proteins

**Fig. 4:**
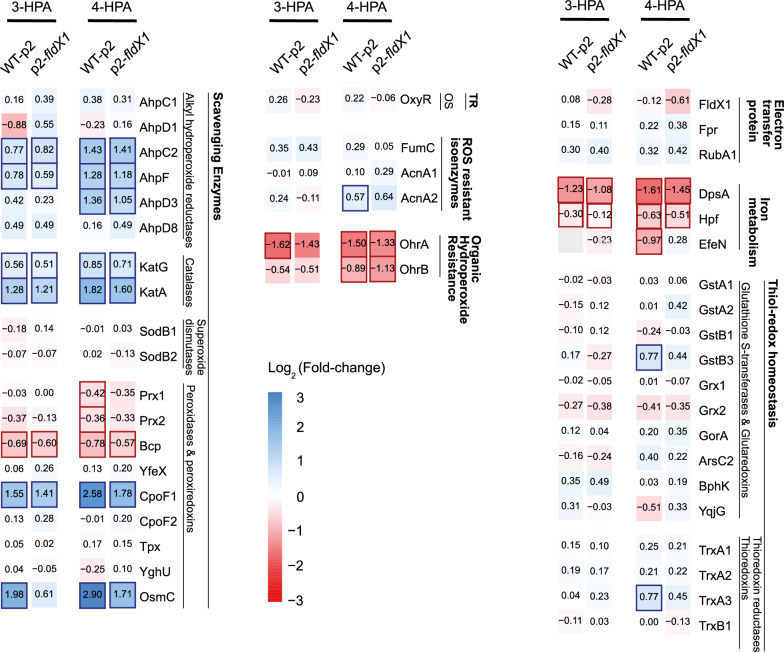
Changes in antioxidant proteins levels of *P. xenovorans* during growth on 3-HPA and 4-HPA. Heatmaps show changes in protein expression levels of cells grown until exponential phase on 3-HPA or 4-HPA compared to glucose-grown cells. Only proteins with variation in the expression levels are represented. Each value represents the average of Log_2_(expression change) of at least four independent experiments. Significant differences are represented as colored border for the corresponding strain. TR: Transcriptional regulator. OS: Oxidative stress. GstA or GstB: Glutathione S-transferase. Grx: Glutaredoxin. GorA: Glutathione peroxidase. ArsC2: Arsenate reductase (glutaredoxin). BphK: Glutathione *S*-transferase (biphenyl degradation pathway). YgjG: Glutathionyl-hydroquinone reductase. TrxA: Thioredoxin. TrxB: Thioredoxin reductase. FumC: Fumarate hydratase class II. AcnA: Aconitate hydratase. AhpC or AhpD: Alkyl hydroperoxide reductase, peroxidase subunit. AhpF: Alkyl hydroperoxide reductase, reductase subunit. Kat: Catalase. SodB: Superoxide dismutase. Prx or Bcp: Peroxiredoxin. YfeX: Dye-decolorizing peroxidase. CpoF: Non-heme chloroperoxidase. Tpx: Thiol peroxidase. YghU: Organic hydroperoxidase. OsmC: Peroxiredoxin. FldX1: Flavodoxin. Fpr: Ferredoxin NADP reductase. RubA1: Rubredoxin. DpsA: Ferritin DPS family DNA-binding protein. Hpf: High potential Fe-S protein. EfeN: Deferrochelatase/peroxidase. OhrA or OhrB: Organic hydroperoxide resistance protein

Figure 4 shows changes of antioxidant-related proteins during growth of p2-*fldX1* and WT-p2 strains in 3-HPA or 4-HPA, compared to growth on glucose. In general, the catabolism of 4-HPA produced more protein changes than 3-HPA compared to glucose grown-cells (Fig. 4), suggesting that growth on 4-HPA may generate an increased oxidative stress response. This tendency is more significant when the proteins with a significant change against control were evaluated. ROS scavenger enzymes AhpC2, AhpF, AhpD3, KatG, KatA, CpoF and OsmC were upregulated in both conditions (3-HPA and 4-HPA), however, higher expression levels were observed in 4-HPA-grown cells. Similarly, an increased downregulation of DpsA, a protein from the ferritin family that protects against oxidative stress, was observed in 4-HPA-grown cells. A significant difference is only observed in 4-HPA-grown cells for the alkyl hydroperoxide reductase system subunit AhpD3, and the organic hydroperoxide resistance protein OhrB, where no significant differences were observed in 3-HPA-grown cells compared to the glucose-grown cells. Overall, a slight repression of stress-related proteins was observed in p2-*fldX1* cells compared to control cells (WT-p2), suggesting that overexpression of FldX1 prevents the antioxidant response due to a protective effect.

A similar protein expression profile related to oxidative response was observed when strains p2-*fldX1* and WT-p2 were compared on the same condition (3-HPA or 4-HPA). For 4-HPA, two thiol-redox homeostasis proteins GstB3 and TrxA3, and a ROS-resistant isoenzyme (AcnA2) were upregulated in strain WT-p2 in comparison to glucose (Fig. 4). Peroxiredoxins Prx1, Prx2, Bcp, and the high-potential iron-sulfur protein Hpf were downregulated in WT-p2. In 3-HPA-grown WT-p2 cells, only two proteins showed a significant difference in expression levels: the peroxiredoxin OsmC was upregulated and the organic hydroperoxide resistance protein A (OhrA) was downregulated in strain WT-p2 grown on 3-HPA. These results suggest that overexpression of the flavodoxin FldX1 leads to a differential response to oxidative stress in both aromatic compounds, observing a higher stress response towards oxidative damage on strain WT-p2 during the growth on 4-HPA.

Several other proteins displayed expression changes. General stress proteins chaperonin GroEL (Bxe_B1569) and a cold-shock protein (Bxe_B2780) were induced during growth of strain p2-*fldX1* on 3-HPA, but not on 4-HPA.The 50S ribosomal protein L36 (Bxe_A0336) were downregulated in the control strain cultured in 3-HPA.

More than 20 membrane proteins, including porins and transporters, showed changes in expression during growth of the strains p2-*fldX1* and WT-p2 in the aromatic compounds. Some of these proteins were induced or repressed in different conditions. Proteins FliC, FlhF, FlgL and FliJ (Bxe_A0103, Bxe_A0132, Bxe_A0151 and Bxe_A0162, respectively), involved in flagella biosynthesis and functionality, were downregulated or “*off*” in the recombinant strain growing on 3-HPA and/or 4-HPA (Additional file 1: Table S4). Fimbria/pilus periplasmic chaperone (Bxe_B2971) was “*on*” in strains p2-*fldX1* and WT-p2 cultured on 3-HPA and 4-HPA. Two major facilitator superfamily (MFS) transporters (Bxe_A3901 and Bxe_B0430), a dicarboxylate periplasmic transporter (Bxe_B0438; DctP family), a sugar ABC transporter (Bxe_A2975), and an OmpC family outer membrane porin were induced only in presence of 3-HPA and 4-HPA, but not on glucose-grown cells (“*on*”) (Additional file 1: Table S3).

### FldX1 decreases gene expression of oxidative stress-related transcripts during growth on HPAs

**Fig. 5:**
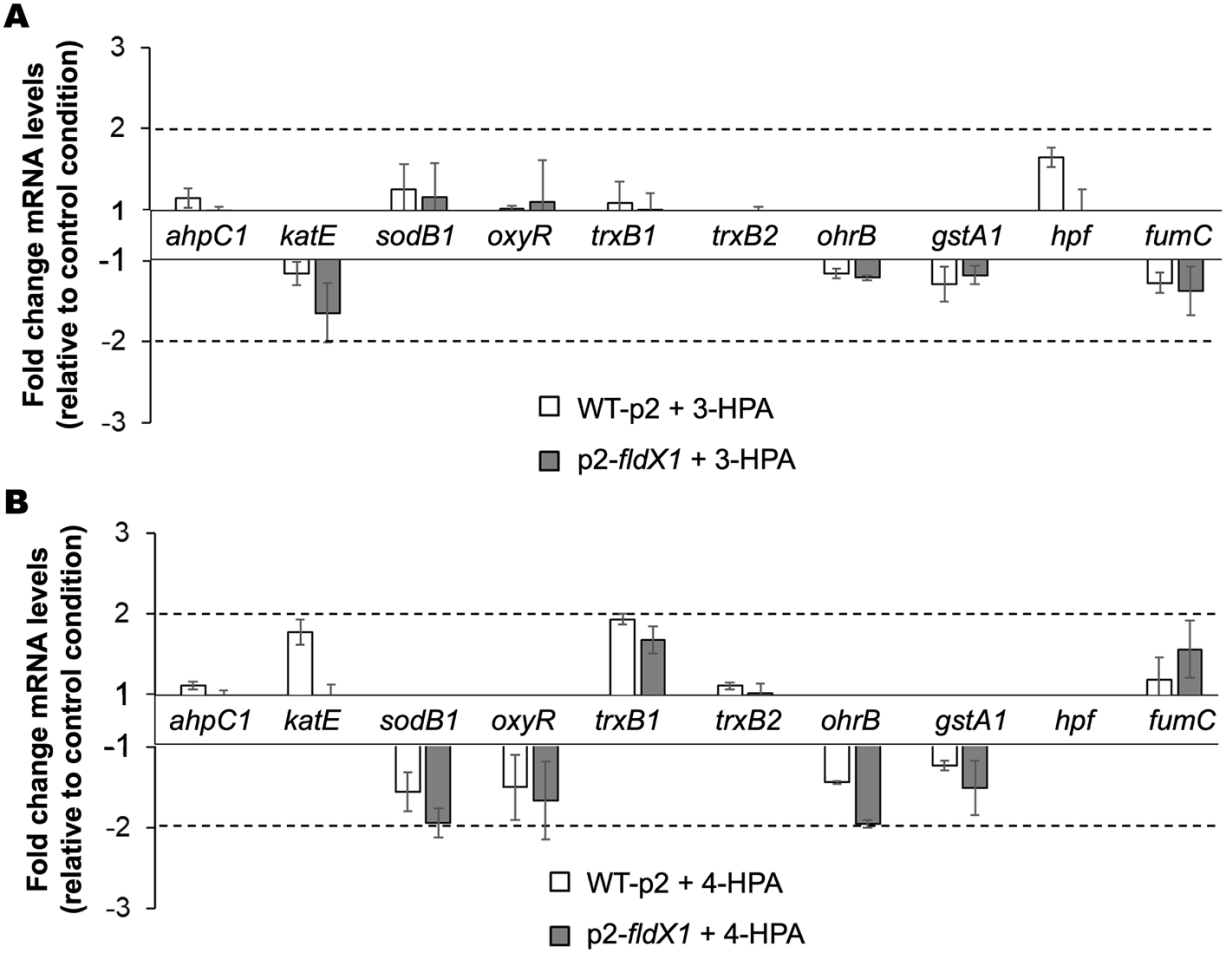
Effects of flavodoxin FldX1 on the expression of antioxidant response genes in *P. xenovorans* during growth on 3-HPA and 4-HPA. Bar plots show changes in expression levels of cells grown until exponential phase on **A,** 3-HPA and **B,** 4-HPA relative to glucose-grown cells. Genes *ftsZ* and *gyrB* were used as reference genes. Each value is an average ± SD of at least three independent experiments

Previously identified proteins associated with oxidative stress mechanisms in strain LB400 [4] were evaluated in detail to compare the oxidative stress response of both strains. To evaluate if the oxidative stress mechanisms were differentially induced in the recombinant strains, gene expression of transcripts involved in the antioxidant response were analyzed by qRT-PCR during growth on 3-HPA and 4-HPA. The genes selected included some of the ROS scavenging enzymes previously identified as part of the antioxidant machinery of strain LB400, such as the alkyl hydroperoxide reductase (*ahpC1*), catalase (*katE*) and superoxide dismutase (*sodB1*), along with the OxyR transcriptional regulator (*oxyR*), two thioredoxin reductases (*trxB1* and *trxB2*), an organic hydroperoxide resistance protein (*ohrB*), a glutathione S-transferase (*gstA1*), a high potential iron-sulfur protein (electron transfer protein; *hpf*) and the ROS resistant isoform of the fumarate hydratase (*fumC*) [4, 29]. No > twofold changes in gene expression levels were observed in cells gown on 3-HPA and 4-HPA compared to glucose (Fig. 5). Nevertheless, strain p2-*fldX1* showed a slight tendency to decrease the expression of specific genes (*i.e., katE* and *fumC* in 3-HPA-grown cells; *katE*, *sodB1, trxB1* and *ohrB* in 4-HPA-grown cells). Interestingly, in both growth conditions, the transcript levels of these genes were higher in the strain WT-p2, whereas lower expression levels were observed in strain p2-*fldX1,* suggesting that FldX1 overexpression might exert an adjuvant role, wherein the expression of these stress-related genes is nonessential*,* or contributing to a more favorable redox environment so their expression is not required.

### FldX1 increases early 4-HPA degradation in soil microcosms assays

To evaluate potential use of the strain overexpressing the flavodoxin FldX1 in bioremediation processes, soil microcosms assays polluted with 4-HPA were carried out. 4-HPA was selected for this study due to the overall catabolic fitness of the strain p2-*fldX1* on this aromatic compound. Moreover, higher ROS levels were observed upon exposure to 4-HPA (Fig. 1).

**Fig. 6:**
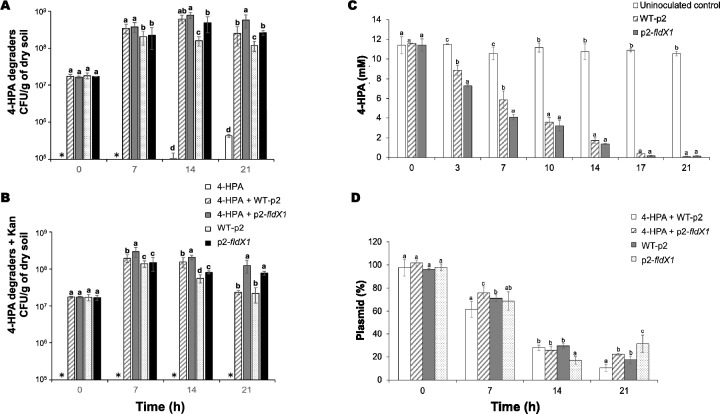
Effect of flavodoxin FldX1 in *P. xenovorans* on 4-HPA degradation in soil microcosms.** A** 4-HPA degraders and (**B**) 4-HPA degraders resistant to kanamycin. (**C**) 4-HPA degradation in soil microcosms**. D** Prevalence of plasmids from *P. xenovorans* recombinant strains in soil microcosms. Significant differences were determined using Fisher's LSD test (p < 0.05). Lowercase letters above the error bars indicate significant differences between treatments. Asterisks indicate value = 0. Each value is an average ± SD of the three replicates of the experiment

An increase in 4-HPA degraders was observed within 14 days of incubation, especially under conditions bioaugmented with strain p2-*fldX1* in the presence of 4-HPA (Fig. 6A, B). All bioaugmented treatments showed a complete 4-HPA degradation at the end of the experiment (Fig. 6C). Notably, strain overexpressing the flavodoxin FldX1 degraded more 4-HPA than the control strain at 3 and 7 days of incubation. In accordance with these results, in presence of 4-HPA (day 21), higher levels of 4-HPA degrading bacteria were observed in soils bioaugmented with strain p2-*fldX1* compared to soils inoculated with WT-p2 (Fig. 6A). In the uninoculated control, colonies were observed from day 14 until the end of the experiment (21 days). Total heterotrophs increased CFU counts per g of dry soil after 14 d incubation, especially in bioaugmented soils (p2-*fldX1* and WT-p2) spiked with 4-HPA (Additional file 1: Fig. S3).

As strains p2-*fldX1* and WT-p2 possess a plasmid with kanamycin resistance as a selection marker, the plasmid was monitored during incubation using M9 minimal medium with the antibiotic and 4-HPA as sole carbon and energy sources (Fig. 6B, D). No growth was detected in the uninoculated condition. In presence of the aromatic compound, the bioaugmented condition with the strain overexpressing the flavodoxin FldX1 exhibited higher growth than the control strain. Interestingly, the recombinant strains p2-*fldX1* and WT-p2 did not show growth differences between them in the absence of 4-HPA. Regarding the plasmid loss, indicated by the decrease in kanamycin resistance, this phenomenon is evident in all the bioaugmented treatments. In presence of 4-HPA, the strain overexpressing the flavodoxin FldX1 showed a lower plasmid loss at days 7 and 21, compared to the control strain. In the absence of the aromatic compound, strain p2-*fldX1* exhibited a higher quantity than strain WT-p2 at day 21. At the end of the assay, only 10 to 30% of plasmid remains (Fig. 6D).

## Discussion

In this study, the effects of the long-chain flavodoxin FldX1 on *P. xenovorans* growth and degradation of aromatic compounds were determined. In a previous report, the long-chain flavodoxin FldX1 from strain LB400 showed a protective role during oxidative stress [29]. Flavodoxin FldX1 improves *P. xenovorans* LB400 tolerance to the oxidizing agents paraquat and H_2_O_2_. Flavodoxin overexpression has been observed to confer enhanced tolerance during oxidative stress conditions in *P. aeruginosa* and tobacco plants [7, 28]. Stress-related proteins associated to ROS-scavenging and detoxification mechanisms are upregulated during exposure of *P. xenovorans* LB400 to (chloro)biphenyls, (chloro)benzoates, and *p*-cymene, indicating that degradation of halogenated and non-halogenated aromatic compounds induces oxidative stress in strain LB400 [8, 10, 14]. In this study, we focused on the degradation of hydroxyphenylacetates as model compounds for aromatic catabolism. *P. xenovorans* LB400 grows on 4-HPA and 3-HPA as sole carbon and energy sources [25]. These benzene-derived aromatic compounds are widely distributed and environmentally relevant since they are products of lignin decomposition [39, 40]. Furthermore, 4-HPA is an industrial pollutant present in wastewater from olive oil production [31]. In this study, we showed that 4-HPA led to an increase in ROS in *P. xenovorans*, specifically the hydroxyl radical. 4-HPA induced an increased accumulation of ROS compared to 3-HPA (Fig. 1).

### Long-chain flavodoxin FldX1 improves bacterial growth of *P. xenovorans* and its catabolic capability in the presence of HPAs

Overexpression of the flavodoxin FldX1 improved the growth of *P. xenovorans* on both 4-HPA and 3-HPA in liquid medium compared to the control strain (Figs. 2A, B). Overexpression of FldX1 also improved degradation of 4-HPA and 3-HPA in *P. xenovorans* (Fig. 2C, D), showing a more efficient degradation of these aromatic compounds by strain p2-*fldX1*. A positive correlation between biomass formation and substrate consumption (4-HPA and 3-HPA) was observed in strains p2-*fldX1* and WT-p2. These results indicate that FldX1 has a positive effect on bacterial growth during hydroxyphenylacetates metabolism. In a previous study, the addition of the antioxidant compound α-tocopherol in soils contaminated with polychlorobiphenyls (PCBs) improved PCB degradation by strain *P. xenovorans* LB400 [2], suggesting that redox imbalance generated by aromatic compounds metabolism can be prevented by the addition of antioxidants, resulting in improved bacterial performance. Similarly, according to the results of this study, flavodoxin FldX1 may be playing a protective role in the antioxidant response, therefore, resulting in an improved growth and hydroxyphenylacetates degradation. Interestingly, strains p2-*fldX1* and WT-p2 grew faster on 4-HPA compared to 3-HPA, which is in accordance with that reported previously, showing that *P. xenovorans* LB400 grows faster on 4-HPA compared to 3-HPA [25]. These results suggest that 3-HPA metabolism may be delayed due to a redox imbalance. The alkyl hydroperoxide reductase AhpC is upregulated during the growth on (chloro)biphenyls, and *p*-cymene of *P. xenovorans* LB400, indicating that H_2_O_2_ is also produced during aromatic compounds degradation [8, 10]. However, in this study, we observed that 4-HPA generates more ROS than 3-HPA and glucose (Fig. 1). The delayed growth on 3-HPA and degradation may be due to different regulation or activity of the catabolic enzymes. Accordingly, in a previous study, we reported that during 3-HPA degradation two catabolic *hmgA* genes are induced, whereas 4-HPA only induced one *hmgA* gene copy [25].

On the other hand, the 4-HPA and 3-HPA are close structural analogues, which may be leading to a differential ROS production during the degradation pathway. The first steps in aerobic aromatic compounds metabolism are mono- or dioxygenation reactions performed by Rieske nonheme iron oxygenases enzymes [41]. However, when an uncoupling of the substrate in their catalytic site occurs, ROS are released by these faulty reactions [2, 42, 43]. The homogentisate pathway has been described in *P. putida* as the central route involved in the catabolism of 3-HPA [44], which uses for the first monooxygenation reaction, the enzyme 3-HPA 6-hydroxylase (MhaAB) [45]. MhaA is a FAD-dependent hydroxylase required to attach a hydroxyl group on 3-HPA. In *P. putida*, the MhaAB enzyme uses 3-HPA as substrate, but does not recognize 4-HPA as substrate [45]. In *P. xenovorans* LB400, both 4-HPA and 3-HPA are degraded via the homogentisate and homoprotocatechuate central pathways [25], which is in accordance with our proteomic results that showed the utilization of both central pathways to metabolize 4-HPA and 3-HPA by the recombinant strains (Fig. 3). However, these results suggest, together with the gene expression analyses (Additional file 1: Fig. S2) that the homogentisate route is predominantly used for both 4-HPA and 3-HPA degradation. Based on these results, we hypothesize that the first hydroxylating reaction of 4-HPA, mediated by MhaAB funneling the homogentisate pathway, may be leading to an increase in ROS formation since 4-HPA is not the preferred substrate. This correlates with our observation that 4-HPA degradation generates more ROS than 3-HPA (Fig. 1). On the other side, in contrast to the oxidation of 3-HPA, the mechanism of 4-HPA oxidation involves an unusual intramolecular migration (so called “NIH shift”) [27, 46, 47]. The enzymatic hydroxylation of aromatic compounds such as 4-HPA and 4-hydroxybenzoate (4-HBA) may induce migration of the original functional group in the C1 position (carboxymethyl group in 4-HPA or carboxyl group in 4-HBA) to the adjacent carbon (1,2-shift) via different reaction mechanisms [27, 47]. We propose that this intramolecular migration (NIH shift) in 4-HPA, which does not occur in 3-HPA, may generate higher ROS levels. Interestingly, recombinant cells overexpressing FldX1, exhibited a diminished stress response in presence of both aromatic compounds, especially in 4-HPA-grown cells. FldX1 may be playing a role as an electronic switch during 4-HPA degradation. FldX1 helps to increase the reducing power by acting as an electron shuttle during the 4-HPA first hydroxylating reaction, thus improving bacterial fitness and oxidative stress response.

**Fig. 7:**
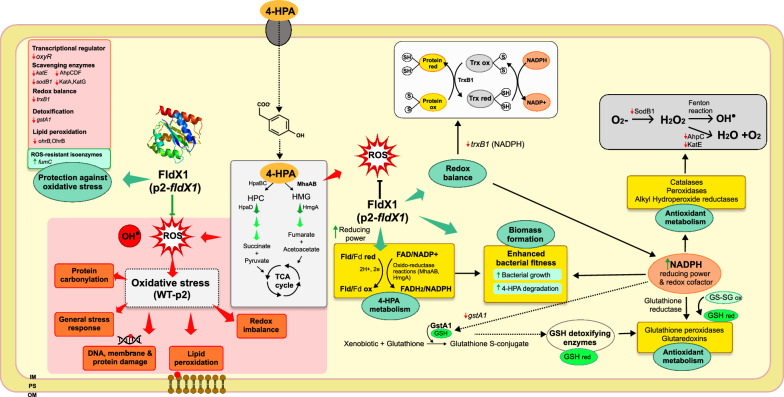
Overview of *P. xenovorans* physiological response during 4-HPA catabolism in presence of FldX1. 4-HPA enters the cell through a transmembrane protein and is catabolized via homoprotocatechuate and homogentisate degradation pathways. The preferred route, homogentisate, generates ROS via the MhaAB enzyme, during an enzymatic reaction facilitated by FldX1, which serves as electron shuttle. This is reflected by a downregulation of the antioxidant response (antioxidant proteins and genes), enhancing bacterial fitness. The long-chain flavodoxin FldX1 acts as electron shuttle between sources of reducing power and metabolic pathways. Green arrows indicate upregulation of genes/proteins, while red arrows indicate downregulation. OM, outer membrane, PS, periplasmic space, IM, inner membrane

FAD-dependent oxidoreductases (i.e., MhaA) are electron-consuming enzymes, requiring FAD conversion into FADH_2_ (Fig. 7). Flavodoxins are isofunctional with ferredoxins, which are widely distributed among organisms, however, flavodoxins are restricted to prokaryotes and some marine algae [48]. Ferredoxins collect reducing equivalents generated during metabolism and distribute them to a number of biosynthetic, regulatory and antioxidant pathways, acting as electron shuttles [49]. Electron shuttling is a common process in oxidation–reduction reactions. Flavodoxins may be acting as “catabolic partners”, *i.e.*, electron carrier proteins that act as electronic switches between cellular sources of reducing power and electron-consuming routes. In this context, FldX1 may be playing a role as a catabolic partner during the first HPA hydroxylating reactions that require a positive redox balance, which results in an enhanced bacterial growth and degradation capabilities and a reduced oxidative stress response. This is reflected in the increased biomass formation and degradation of HPAs in presence of FldX1, compared to the control strain, as well as on the proteomic and transcriptional analyses (Fig. 7). These results showed a protective effect of FldX1 against oxidative stress generated by the catabolism of these aromatic compounds.

### FldX1 prevents oxidative stress response generated by aromatic degradation

Previous proteomic and transcriptional analyses in LB400 revealed the upregulation of several antioxidant response proteins during 4-HPA and 3-HPA catabolism, including Ahp, Sod, Kat, Ohr, along with proteins involved in enhancing reducing power (Hpf, Trx) [50]. In this study, the antioxidant response in presence of FldX1 during growth on 4-HPA and 3-HPA was evaluated through proteomic and transcriptional analyses (Figs. 4 and 5). ROS-scavenging enzymes (alkyl hydroperoxide reductases, catalases, superoxide dismutases, peroxidases and peroxiredoxins) were slightly upregulated during growth on 4-HPA*.* Upregulated proteins included alkyl hydroperoxide reductases AhpC2, AhpF and AhpD3, the catalase KatA, peroxidases Bcp and CpoF1, and peroxiredoxins Prx1 and Prx2. These scavenging enzymes participate in H_2_O_2_ detoxification during oxidative stress. The AhpC protein was also upregulated in the wild-type strain *P. xenovorans* LB400 growing on biphenyl and *p*-cymene [8, 10]. These results suggest that 4-HPA degradation produces more ROS than 3-HPA, which correlates with ROS formation assays (Fig. 1). Overall, we observed that cells overexpressing FldX1 prevented the expression of stress-related proteins, suggesting that FldX1 attenuates the oxidative stress response during growth on 3-HPA and 4-HPA (Fig. 4). This correlates with transcriptional analysis, in which a downregulation of stress-related genes was observed in cells overexpressing FldX1 (*i.e.*, *oxyR*, *sodB1*, *trxB1*, *ohrB*, *gstA1*). This was observed in presence of 4-HPA as sole carbon source, a growth condition that in general exhibited more changes in both proteomic and transcriptional analyses compared to 3-HPA. This phenomenon may be correlated with a higher production of ROS found in cells exposed to 4-HPA (Fig. 1), in which the stress response appears to be amplified, in contrast to the 3-HPA condition. The protective effect of the long-chain flavodoxins against an oxidative stress condition has been reported. Overexpression of the long-chain flavodoxin IsiB from the cyanobacterium *Anabaena variabilis* PCC7119 in *E. coli*, *P. fluorescens*, and *E. meliloti*, conferred protection against H_2_O_2_, paraquat, and atrazine [9]. Similarly, the long-chain flavodoxin FldP from *P. aeruginosa* protects bacterial cells from oxidative stress, thereby expanding its capabilities to thrive in hostile environments [7]. However, the mechanism by which the long-chain flavodoxin exerts a protective effect has not been elucidated.

Our proteomic results showed that the protein OhrB (Bxe_B2843) was slightly downregulated in p2-*fldX1* cultured in 4-HPA, compared to glucose, similar to that observed in the qRT-PCR analysis. The Ohr family (organic hydroperoxide resistance) plays a central role in the bacterial response to stress induced by organic hydroperoxides. Ohr peroxidase requires reduction of its disulfide group upon catalytic activity on the organic hydroperoxide [51]. Downregulation observed in recombinant cells overexpressing FldX1 suggests that the long-chain flavodoxin may be playing a key role in distribution of reducing power to prevent oxidative stress and cellular damage (i.e., cell membranes). ROS cause direct or indirect damage to DNA, proteins, and cell membranes, producing mutagenesis, lipoperoxidation, and protein carbonylation and oxidation (Fig. 7). FldX1 decreased protein carbonylation upon exposure to the oxidizing agent hydrogen peroxide [29]. An upregulation of the Ohr protein was observed in LB400 upon exposure to paraquat and hydrogen peroxide [4]. Therefore, in addition to contributing to the catabolism of 4-HPA as an electron shuttle, FldX1 also distributes reducing power, shuttling electrons resulting in an overall improved bacterial fitness. Furthermore, FldX1 enhanced *P. xenovorans* survival upon exposure to low and high concentrations of paraquat [29].

Cell survival relies on the enzyme activity of superoxide dismutase, catalase, and glutathione/glutaredoxin recycling systems, and the availability of antioxidants (*e.g.*, GSH) to scavenge ROS produced by endogenous reactions or environmental insults [3]. The glutathione S-transferase GstB3 (Bxe_B1775) and the thioredoxin reductase TrxA3 (Bxe_B1336), enzymes involved in thiol-redox homeostasis, showed significant differences in p2-*fldX1* cells grown in 4-HPA. These enzymes play a key role in the maintenance of cellular redox state through GSH and NADPH as reductants. The reducing power NADPH is necessary to fuel detoxifying enzymes (typically dependent on reduced glutathione GSH). In *P. xenovorans* LB400, carbon intermediates are metabolized via the Entner–Doudoroff, pentose-phosphate, and lower Embden–Meyerhoff–Parnas pathways, which produce more reducing power through NADPH synthesis than the classical Embden–Meyerhoff–Parnas glycolysis [52]. This indicates that LB400 synthesizes more NADPH molecules as reducing power sources for consuming processes, such as oxidative stress response. A similar phenomenon occurs in *P. putida*. An increase in the NADPH pool in *P. putida* provides a robust machinery involving strong redox transactions towards the metabolization of novel xenobiotic substrates [53]. *P. putida* can tolerate high levels of oxidative stress by rerouting carbon metabolism towards NADPH formation, as a key source of reducing power to fuel the glutathione system [54]. The most common mechanism to manage oxidative stress involves the action of ROS detoxifying enzymes (*e.g.*, catalases, peroxidases, and hydroperoxide reductases). The corresponding reactions of these antioxidant enzymes eventually consume metabolic NADPH, which provides the reductive molecule to counteract the toxic effects of ROS, *e.g.*, via reduced glutathione [55]. The glutathione cycle, which connects the reduced (GSH) and the oxidized (GS–SG) forms of the thiol, acts as a preferred reductant of ROS via the glutathione peroxidases and glutaredoxins enzymes (Fig. 7). The GSH pool is restored by using NADPH as a reductant [56].

**Fig. 8:**
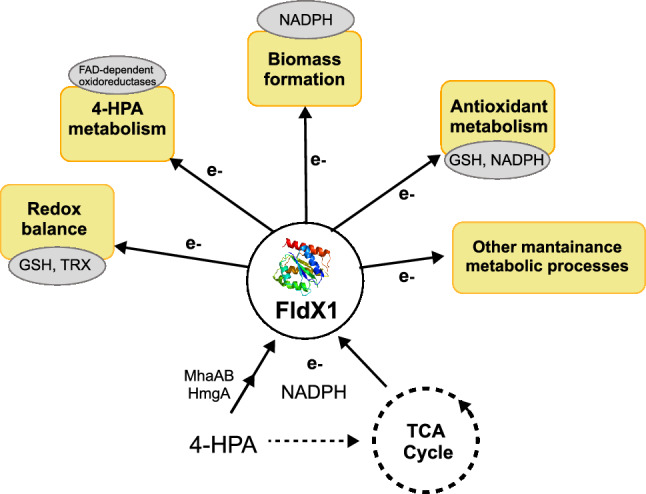
Outline of the long-chain flavodoxin FldX1 effects on *P. xenovorans* physiology. Major improvements were observed in maintaining redox balance, 4-HPA degradation, biomass formation and antioxidant metabolism via electron shuttling from metabolic pathways (TCA cycle and 4-HPA)

In *P. xenovorans*, the overexpression of the long-chain flavodoxin FldX1 enables the gathering of reducing equivalents, generated from the central metabolism (carbon) and the 4-HPA metabolism, and delivers them to several metabolic and regulatory processes (Fig. 8), improving bacterial fitness and antioxidant response.

### Bioremediation potential evaluation of FldX1 using 4-HPA in soil microcosms

In this study, we observed that FldX1 provided enhanced bacterial fitness during hydroxyphenylacetates metabolism, especially with 4-HPA. This was revealed mainly by improving growth on 4-HPA and its degradation capabilities, along with a downregulation of the oxidative stress response in *P. xenovorans* p2-*fldX1*. To evaluate the potential use in bioremediation processes of the strain p2-*fldX1* in a natural environment, soil microcosms assays were performed with soil supplemented with 4-HPA. Within 7 days of incubation, an improved 4-HPA degradation was observed in soils inoculated with p2-*fldX1* cells, in comparison with soils enriched with the control strain, which correlates with the in vitro degradation assays. After a week, the 4-HPA-degraders increased, especially in conditions inoculated with p2-*fldX1.* All bioaugmented conditions were capable of completely catabolizing the aromatic compound (Fig. 6)*.* The presence of microorganisms capable to degrade 4-HPA in a soil matrix was expected since 4-HPA is an intermediary of lignin degradation pathway [39, 40], which is part of the lignocellulose, the most abundant polymer on Earth [57, 58].

To analyze the recombinant plasmid loss during the soil microcosm experiment, the decrease of the capability to grow in presence of the antibiotic kanamycin, the plasmid selection marker, was assessed. Nevertheless, this approach is not an absolute indicator of the presence of the *P. xenovorans* recombinant strains, because horizontal gene transfer has been described in soil from the strains employed for bioaugmentation to the native microbial community [59]. In all treatments, plasmid loss was between 70 to 90%. Interestingly, at specific incubation times, the treatments inoculated with the strain p2-*fldX1* showed a higher plasmid prevalence compared to those bioaugmented with the control strain. Therefore, the plasmid p2-*fldX1* conferred a physiological advantage to *P. xenovorans* in soils polluted with 4-HPA. Plasmids and other Darwinian mobile genetic elements may improve the fitness of bacteria, conferring several characteristics, including the capability to degrade or synthesize a compound, tolerate metals, and resistance to antibiotics [60]. Nevertheless, when the microorganism is in a non-selective environment, where the characteristic conferred by the plasmid vector is not needed, the plasmid may constitute an unnecessary metabolic burden. Due to this phenomenon, bacterial plasmid loss through generations has been widely observed [61, 62]. The only physiological advantage that confers the plasmid vector of the control strain is kanamycin resistance. Considering that the soil lacks this antibiotic, the plasmid loss was expected. Interestingly, strain p2-*fldX1* retained higher levels of plasmid than strain WT-p2 under selective (4-HPA) and non-selective (absence of 4-HPA) conditions.

Overall, these results revealed that the recombinant strain p2-*fldX1* improves 4-HPA degradation in liquid cultures and soil microcosms, probably due to its protective effect against oxidative stress generated during 4-HPA metabolism. FldX1 as an electron shuttle is able to deliver reducing power to several metabolic processes including the antioxidant stress response (Fig. 8). This is an extremely valuable feature for bioremediation strategies, since bacterial cells are constantly exposed to multiple stressors, in which FldX1 is also enhancing degradation capabilities. Thus, overexpression of FldX1 has a high potential for bioremediation processes of aromatic compounds.

## Conclusions

Previous studies showed that aerobic aromatic catabolism by the model bacterium *P. xenovorans* LB400 leads to a general stress response, including oxidative stress/damage. In this study, the effects of the long-chain flavodoxin FldX1 on *P. xenovorans* LB400 fitness during growth in two aromatic compounds (4-HPA and 3-HPA) as sole carbon and energy sources were assessed. The overexpression of FldX1 confers the capability to grow faster on 4-HPA and 3-HPA, and to degrade them more efficiently in liquid culture. A higher performance was observed especially in 4-HPA-grown cells. Proteomic and gene expression analyses showed the overall antioxidant effects of the flavodoxin FldX1 in the cell, suggesting that delivery of electrons towards several cellular processes, including the antioxidant response and catabolism of aromatic compounds, may explain the enhanced bacterial fitness observed. Bioaugmentation with the strain that overexpress FldX1 showed higher 4-HPA degradation in soil microcosms than inoculation with strain WT-p2. These results demonstrate that the recombinant strain *P. xenovorans* p2-*fldX1* is an attractive candidate for bioremediation processes of aromatic compounds.

### Supplementary Information

**Additional file 1: Figure S1.** Visualization of shared and unique proteins in all analyzed strains and conditions. Bar charts on top of each column represent the number of proteins that are shared in the indicated set of conditions (lower part of the diagram). Total number of proteins per condition is indicated by the lower left bar chart. **Figure S2. **Effects of the long-chain flavodoxin FldX1 in expression of genes encoding dioxygenases of *P. xenovorans* during growth on 3-HPA and 4-HPA. Changes in expression levels of cells grown until exponential phase are shown in **A,** 3-HPA and **B,** 4-HPA relative to glucose as sole carbon source. Genes *ftsZ* and *gyrB* were used as reference genes. Each value is an average ± SD of at least three independent experiments. Significant differences between groups were evaluated using a two-tailed independent t-test (p < 0.05) on log fold change values (asterisk). **Figure S3. **Total heterotrophs in soil microcosms. Total heterotrophs were determined in LB medium and incubated for 24 h at 30ºC, using *P. xenovorans* recombinant strains amended with 4-HPA. Corresponding controls are indicated. Significant differences were determined with LSD Fisher test (p < 0.05). Lower case letters under error bars indicate significant differences between treatment at each time. Significant differences were determined using Fisher's LSD test (p < 0.05). Asterisks indicate value = 0. Each value is the mean ± SD of all replicates of the experiment. **Table S1.** Primers used in this study. **Table S2.** Please visit https://zenodo.org/records/10814965. **Table S3.** Proteins detected during growth on 3-HPA and 4-HPA but not in the control condition (“on”). **Table S4.** Proteins detected in the control condition (glucose) but not during growth on 3-HPA and 4-HPA (“off”).

## Data Availability

All data generated or analyzed during this study are included in this published article.

## References

[1] Cabiscol E, Tamarit J, Ros J (2000). Oxidative stress in bacteria and protein damage by reactive oxygen species. Int Microbiol.

[2] Ponce BL, Latorre VK, González M, Seeger M (2011). Antioxidant compounds improved PCB-degradation by *Burkholderia xenovorans* strain LB400. Enzyme Microb Technol.

[3] Imlay JA (2013). The molecular mechanisms and physiological consequences of oxidative stress: lessons from a model bacterium. Nat Rev Microbiol.

[4] Méndez V, Rodríguez-Castro L, Durán RE, Padrón G, Seeger M (2022). The OxyR and SoxR transcriptional regulators are involved in a broad oxidative stress response in *Paraburkholderia xenovorans* LB400. Biol Res.

[5] Khademian M, Imlay JA (2017). *Escherichia coli* cytochrome c peroxidase is a respiratory oxidase that enables the use of hydrogen peroxide as a terminal electron acceptor. Proc Natl Acad Sci USA.

[6] Price-Whelan A, Dietrich LEP, Newman DK (2006). Rethinking “secondary” metabolism: physiological roles for phenazine antibiotics. Nat Chem Biol.

[7] Moyano AJ, Tobares RA, Rizzi YS, Krapp AR, Mondotte JA, Bocco JL (2014). A long-chain flavodoxin protects *Pseudomonas aeruginosa* from oxidative stress and host bacterial clearance. PLoS Genet.

[8] Response to(chloro)biphenyls of the polychlorobiphenyl-degrader *Burkholderia xenovoran*s LB400 involves stress proteins also induced by heat shock and oxidative stress. FEMS Microbiology Letters; 2007. 10.1111/j.1574-6968.2006.00554.x 17166226

[9] Coba de la Peña T, Redondo FJ, Fillat MF, Lucas MM, Pueyo JJ (2013). Flavodoxin overexpression confers tolerance to oxidative stress in beneficial soil bacteria and improves survival in the presence of the herbicides paraquat and atrazine. J Appl Microbiol.

[10] Agulló L, Romero-Silva MJ, Domenech M, Seeger M (2017). *p*-Cymene promotes its catabolism through the *p*-cymene and the *p*-cumate pathways, activates a stress response and reduces the biofilm formation in *Burkholderia xenovorans* LB400. PLoS ONE.

[11] Sen A, Imlay JA (2021). How microbes defend themselves from incoming hydrogen peroxide. Front Immunol.

[12] Kim YH, Cho K, Yun S-H, Kim JY, Kwon K-H, Yoo JS (2006). Analysis of aromatic catabolic pathways in *Pseudomonas putida* KT 2440 using a combined proteomic approach: 2-DE/MS and cleavable isotope-coded affinity tag analysis. Proteomics.

[13] Tam LT, Eymann C, Albrecht D, Sietmann R, Schauer F, Hecker M (2006). Differential gene expression in response to phenol and catechol reveals different metabolic activities for the degradation of aromatic compounds in *Bacillus subtilis*. Environ Microbiol.

[14] Martínez P, Agulló L, Hernández M, Seeger M (2007). Chlorobenzoate inhibits growth and induces stress proteins in the PCB-degrading bacterium *Burkholderia xenovorans* LB400. Arch Microbiol.

[15] Pieper DH, Seeger M (2008). Bacterial metabolism of polychlorinated biphenyls. J Mol Microbiol Biotechnol.

[16] Lin J (2017). Stress responses of *Acinetobacter* strain Y during phenol degradation. Arch Microbiol.

[17] Gibson DT, Parales RE (2000). Aromatic hydrocarbon dioxygenases in environmental biotechnology. Curr Opin Biotechnol.

[18] Imbeault NY, Powlowski JB, Colbert CL, Bolin JT, Eltis LD (2000). Steady-state kinetic characterization and crystallization of a polychlorinated biphenyl-transforming dioxygenase. J Biol Chem.

[19] Patrauchan MA, Florizone C, Eapen S, Gómez-Gil L, Sethuraman B, Fukuda M (2008). Roles of ring-hydroxylating dioxygenases in styrene and benzene catabolism in *Rhodococcus jostii* RHA1. J Bacteriol.

[20] Seeger M, Timmis KN, Hofer B (1995). Conversion of chlorobiphenyls into phenylhexadienoates and benzoates by the enzymes of the upper pathway for polychlorobiphenyl degradation encoded by the bph locus of *Pseudomona*s sp. strain LB400. Appl Environ Microbiol.

[21] Seeger M, Zielinski M, Timmis KN, Hofer B (1999). Regiospecificity of dioxygenation of di- to pentachlorobiphenyls and their degradation to chlorobenzoates by the bph-encoded catabolic pathway of *Burkholderia* sp. strain LB400. Appl Environ Microbiol.

[22] Seeger M, Cámara B, Hofer B (2001). Dehalogenation, denitration, dehydroxylation, and angular attack on substituted biphenyls and related compounds by a biphenyl dioxygenase. J Bacteriol.

[23] Seeger M, González M, Cámara B, Muñoz L, Ponce E, Mejías L (2003). Biotransformation of natural and synthetic isoflavonoids by two recombinant microbial enzymes. Appl Environ Microbiol.

[24] Chain PSG, Denef VJ, Konstantinidis KT, Vergez LM, Agulló L, Reyes VL (2006). *Burkholderia xenovorans* LB400 harbors a multi-replicon, 9.73-Mbp genome shaped for versatility. Proc Natl Acad Sci USA.

[25] Méndez V, Agulló L, González M, Seeger M (2011). The homogentisate and homoprotocatechuate central pathways are involved in 3- and 4-hydroxyphenylacetate degradation by *Burkholderia xenovorans* LB400. PLoS ONE.

[26] Chirino B, Strahsburger E, Agulló L, González M, Seeger M (2013). Genomic and functional analyses of the 2-aminophenol catabolic pathway and partial conversion of its substrate into picolinic acid in *Burkholderia xenovorans* LB400. PLoS ONE.

[27] Romero-Silva MJ, Méndez V, Agulló L, Seeger M (2013). Genomic and functional analyses of the gentisate and protocatechuate ring-cleavage pathways and related 3-hydroxybenzoate and 4-hydroxybenzoate peripheral pathways in *Burkholderia xenovorans* LB400. PLoS ONE.

[28] Zurbriggen MD, Tognetti VB, Fillat MF, Hajirezaei M-R, Valle EM, Carrillo N (2008). Combating stress with flavodoxin: a promising route for crop improvement. Trends Biotechnol.

[29] Rodríguez-Castro L, Méndez V, Durán RE, Seeger M (2019). Long-chain flavodoxin FldX1 improves *Paraburkholderia xenovorans* LB400 tolerance to oxidative stress caused by paraquat and H_2_O_2_. PLoS ONE.

[30] Sancho J (2006). Flavodoxins: sequence, folding, binding, function and beyond. Cell Mol Life Sci.

[31] Hawumba JF, Brözel VS, Theron J (2007). Cloning and characterization of a 4-hydroxyphenylacetate 3-hydroxylase from the thermophile *Geobacillus* sp. PA-9. Curr Microbiol.

[32] Cárdenas F (2015). Biotransformation and effect of flavonoids on oxidative stress during the degradation of aromatic compounds in *Burkholderia xenovorans* LB400.

[33] Mücke P-A, Maaß S, Kohler TP, Hammerschmidt S, Becher D (2020). Proteomic adaptation of *Streptococcus pneumoniae* to the human antimicrobial peptide LL-37. Microorganisms..

[34] Tyanova S, Temu T, Cox J (2016). The MaxQuant computational platform for mass spectrometry-based shotgun proteomics. Nat Protoc.

[35] Tyanova S, Temu T, Sinitcyn P, Carlson A, Hein MY, Geiger T (2016). The Perseus computational platform for comprehensive analysis of (prote)omics data. Nat Methods.

[36] Perez-Riverol Y, Csordas A, Bai J, Bernal-Llinares M, Hewapathirana S, Kundu DJ (2019). The PRIDE database and related tools and resources in 2019: improving support for quantification data. Nucleic Acids Res.

[37] Hellemans J, Mortier G, De Paepe A, Speleman F, Vandesompele J (2007). qBase relative quantification framework and software for management and automated analysis of real-time quantitative PCR data. Genome Biol.

[38] Patrauchan MA, Parnell JJ, McLeod MP, Florizone C, Tiedje JM, Eltis LD (2011). Genomic analysis of the phenylacetyl-CoA pathway in *Burkholderia xenovorans* LB400. Arch Microbiol.

[39] Deangelis KM, D’Haeseleer P, Chivian D, Fortney JL, Khudyakov J, Simmons B (2011). Complete genome sequence of “*Enterobacter lignolyticus*” SCF1. Stand Genomic Sci.

[40] Guo W, Zhou W, Zhou H, Chen X (2019). Characterization of enzymatic properties of two novel enzymes, 3,4-dihydroxyphenylacetate dioxygenase and 4-hydroxyphenylacetate 3-hydroxylase, from *Sulfobacillus acidophilus* TPY. BMC Microbiol.

[41] Seo J-S, Keum Y-S, Li QX (2009). Bacterial degradation of aromatic compounds. Int J Environ Res Public Health.

[42] Pérez-Pantoja D, Nikel PI, Chavarría M, de Lorenzo V (2013). Endogenous stress caused by faulty oxidation reactions fosters evolution of 2,4-dinitrotoluene-degrading bacteria. PLoS Genet.

[43] Kim J, Park W (2014). Oxidative stress response in *Pseudomonas putida*. Appl Microbiol Biotechnol.

[44] Arias-Barrau E, Olivera ER, Luengo JM, Fernández C, Galán B, García JL (2004). The homogentisate pathway: a central catabolic pathway involved in the degradation of L-phenylalanine, L-tyrosine, and 3-hydroxyphenylacetate in *Pseudomonas putida*. J Bacteriol.

[45] Arias-Barrau E, Sandoval A, Naharro G, Olivera ER, Luengo JM (2005). A two-component hydroxylase involved in the assimilation of 3-hydroxyphenyl acetate in *Pseudomonas putida*. J Biol Chem.

[46] Fairley DJ, Boyd DR, Sharma ND, Allen CCR, Morgan P, Larkin MJ (2002). Aerobic metabolism of 4-hydroxybenzoic acid in *Archaea* via an unusual pathway involving an intramolecular migration (NIH shift). Appl Environ Microbiol.

[47] Zhao H, Xu Y, Lin S, Spain JC, Zhou N-Y (2018). The molecular basis for the intramolecular migration (NIH shift) of the carboxyl group during *para*-hydroxybenzoate catabolism. Mol Microbiol.

[48] Lodeyro AF, Ceccoli RD, Pierella Karlusich JJ, Carrillo N (2012). The importance of flavodoxin for environmental stress tolerance in photosynthetic microorganisms and transgenic plants. Mechanism, evolution and biotechnological potential. FEBS Lett.

[49] Pierella Karlusich JJ, Lodeyro AF, Carrillo N (2014). The long goodbye: the rise and fall of flavodoxin during plant evolution. J Exp Bot.

[50] Méndez V (2017). Mecanismos moleculares de la respuesta adaptativa de estrés oxidativo de *Burkholderia xenovorans* LB400 frente a compuestos oxidantes y al metabolismo de compuestos aromáticos.

[51] Cussiol JRR, Alegria TGP, Szweda LI, Netto LES (2010). Ohr (organic hydroperoxide resistance protein) possesses a previously undescribed activity, lipoyl-dependent peroxidase. J Biol Chem.

[52] Alvarez-Santullano N, Villegas P, Mardones MS, Durán RE, Donoso R, González A (2021). Genome-wide metabolic reconstruction of the synthesis of polyhydroxyalkanoates from sugars and fatty acids by *Burkholderia sensu lato* species. Microorganisms..

[53] Akkaya Ö, Pérez-Pantoja DR, Calles B, Nikel PI, de Lorenzo V (2018). The Metabolic Redox Regime of *Pseudomonas putida* tunes its evolvability toward novel xenobiotic substrates. MBio.

[54] Nikel PI, Fuhrer T, Chavarría M, Sánchez-Pascuala A, Sauer U, de Lorenzo V (2021). Reconfiguration of metabolic fluxes in *Pseudomonas putida* as a response to sub-lethal oxidative stress. ISME J.

[55] Tamburro A, Robuffo I, Heipieper HJ, Allocati N, Rotilio D, Di Ilio C (2004). Expression of glutathione S-transferase and peptide methionine sulphoxide reductase in *Ochrobactrum anthropi* is correlated to the production of reactive oxygen species caused by aromatic substrates. FEMS Microbiol Lett.

[56] Vašková J, Kočan L, Vaško L, Perjési P (2023). Glutathione-related enzymes and proteins: a review. Molecules.

[57] Deangelis KM, Sharma D, Varney R, Simmons B, Isern NG, Markilllie LM (2013). Evidence supporting dissimilatory and assimilatory lignin degradation in *Enterobacter lignolyticus* SCF1. Front Microbiol.

[58] Orellana R, Chaput G, Markillie LM, Mitchell H, Gaffrey M, Orr G (2017). Multi-time series RNA-seq analysis of *Enterobacter lignolyticus* SCF1 during growth in lignin-amended medium. PLoS ONE.

[59] Bravo G, Vega-Celedón P, Gentina JC, Seeger M (2020). Bioremediation by *Cupriavidus metallidurans* strain MSR33 of mercury-polluted agricultural soil in a rotary drum bioreactor and its effects on nitrogen cycle microorganisms. Microorganisms.

[60] van Dijk B, Buffard P, Farr AD, Giersdorf F, Meijer J, Dutilh BE (2023). Identifying and tracking mobile elements in evolving compost communities yields insights into the nanobiome. ISME Commun.

[61] Smith MA, Bidochka MJ (1998). Bacterial fitness and plasmid loss: the importance of culture conditions and plasmid size. Can J Microbiol.

[62] Carroll AC, Wong A (2018). Plasmid persistence: costs, benefits, and the plasmid paradox. Can J Microbiol.

