# Supplementary material for: The long-chain flavodoxin FldX1 improves the biodegradation of 4-hydroxyphenylacetate and 3-hydroxyphenylacetate and counteracts the oxidative stress associated to aromatic catabolism in Paraburkholderia xenovorans
Source: Biol Res. 2024 Apr 1;57:12. doi: 10.1186/s40659-024-00491-4 (PMC10983741; doi:10.1186/s40659-024-00491-4)
Supplement: Supplementary file 1 — Additional file 1: Figure S1. Visualization of shared and unique proteins in all analyzed strains and conditions. Bar charts on top of each column represent the number of proteins that are shared in the indicated set of conditions (lower part of the diagram). Total number of proteins per condition is indicated by the lower left bar chart. Figure S2. Effects of the long-chain flavodoxin FldX1 in expression of genes encoding dioxygenases of P. xenovorans during growth on 3-HPA and 4-HPA. Changes in expression levels of cells grown until exponential phase are shown in A, 3-HPA and B, 4-HPA relative to glucose as sole carbon source. Genes ftsZ and gyrB were used as reference genes. Each value is an average ± SD of at least three independent experiments. Significant differences between groups were evaluated using a two-tailed independent t-test (p < 0.05) on log fold change values (asterisk). Figure S3. Total heterotrophs in soil microcosms. Total heterotrophs were determined in LB medium and incubated for 24 h at 30ºC, using P. xenovorans recombinant strains amended with 4-HPA. Corresponding controls are indicated. Significant differences were determined with LSD Fisher test (p < 0.05). Lower case letters under error bars indicate significant differences between treatment at each time. Significant differences were determined using Fisher's LSD test (p < 0.05). Asterisks indicate value = 0. Each value is the mean ± SD of all replicates of the experiment. Table S1. Primers used in this study. Table S2. Please visit https://zenodo.org/records/10814965. Table S3. Proteins detected during growth on 3-HPA and 4-HPA but not in the control condition (“on”). Table S4. Proteins detected in the control condition (glucose) but not during growth on 3-HPA and 4-HPA (“off”). [file 40659_2024_491_MOESM1_ESM.docx]

**Additional file Figures**

**Additional file 1: Figure S1. Visualization of shared and unique proteins in all analyzed strains and conditions**. Bar charts on top of each column represent the number of proteins that are shared in the indicated set of conditions (lower part of the diagram). Total number of proteins per condition is indicated by the lower left bar chart.

**Additional file 1: Figure S2.** **Effects of the long-chain flavodoxin FldX1 in expression of genes encoding dioxygenases of *P. xenovorans* during growth on 3-HPA and 4-HPA.** Changes in expression levels of cells grown until exponential phase are shown in **A,** 3-HPA, and **B,** 4-HPA relative to glucose as sole carbon source. Genes *ftsZ* and *gyrB* were used as reference genes. Each value is an average ± SD of at least three independent experiments. Significant differences between groups were evaluated using a two-tailed independent t-test (p<0.05) on log fold change values (asterisk).

**Additional file 1: Figure S3. Total heterotrophs in soil microcosms.** Total heterotrophs were determined in LB medium and incubated for 24 h at 30ºC, using *P. xenovorans* recombinant strains amended with 4-HPA. Corresponding controls are indicated. Significant differences were determined with LSD Fisher test (p<0.05). Lower case letters under error bars indicate significant differences between treatment at each time. Significant differences were determined using Fisher's LSD test (p<0.05). Asterisks indicate value = 0. Each value is the mean ± SD of all replicates of the experiment.

**Additional file Tables**

| **Additional file 1: Table S1. Primers used in this study** | | | |  |
| --- | --- | --- | --- | --- |
| **Gene** | **Locus tag** | **Primer** | **Sequence 5´-3´** | **Reference** |
| *ahpC1* | Bxe_A2309 | AhpA2309Fw | GCGTCGACAACGAATTCGTG | [32] |
|  |  | AhpA2309Rv | TCGATCAGCTCGCCTTTCAC |  |
| *katE* | Bxe_B1215 | KatB1215Fw | TCATCGAGGAAGCGGACGAA | [32] |
|  |  | KatB1215Rv | TGTCCGGATTGCGATTGAGC |  |
| *sodB1* | Bxe_A0769 | SodA1769Fw | GCGGCTCAAGTGTGGAATCA | [32] |
|  |  | SodA1769Rv | CTGCGGTCTTGGCGA ATTCT |  |
| *oxyR* | Bxe_A3987 | OxA39Fw | GAAGCGTGTTTCGTCAGCCA | [4] |
|  |  | OxA39Rv | TTCGAGGACACGTTGAGCTT |  |
| *txrB1* | Bxe_A3442 | A3442TrFw | CCCATGAACGCTTCTTCCGA | [4] |
|  |  | A3442TrRv | ACCACATTCACACGGCAAAG |  |
| *txrB2* | Bxe_A3962 | A3962TxFw | GACCGGTGTCGATTGGTGTT | [4] |
|  |  | A3962TxRv | ATAAACCCGTGCTGCTCGATT |  |
| *ohrB* | Bxe_B2843 | OhrB2843Fw | CCCGGCGACAAACTTCATTG | [4] |
|  |  | OhrB2843Rv | GGCGGTGTGTCGGATAAT |  |
| *gst* | Bxe_A0624 | GstA0624Fw | GCGACAGCGTATTCCAGGTATT | [4] |
|  |  | GstA0624Rv | CTGGTGCCCCAGAATGTCTG |  |
| *hpf* | Bxe_A4333 | HpfFw | CGAGCAAAGTCGACAAAGCG | [4] |
|  |  | HpfRv | TATATGCGCTGCACCAACCC |  |
| *fumC* | Bxe_A1038 | FumA1038Fw | CGTACGAATGGAGCGTGACA | [32] |
|  |  | FumA1038Rv | ATGAGTTCGGGCGATTGCTT |  |
| *hmgA1* | Bxe_A2725 | BxeA2725_HmgA1Fw | AAGTTCCAGGGCAACCTATG | [50] |
|  |  | BxeA2725_HmgA1Rv | GGCGGAAAGATCACGAAGT |  |
| *hmgA2* | Bxe_A3900 | BxeA3900_HmgA2Fw | TCGACATTGCCACCGAAA | [50] |
|  |  | BxeA3900_HmgA2Rv | ATGCGGCGTGAGGAAAT |  |
| *hpaD* | Bxe_B2031 | BxeB2031_HpaDFw | CCGAAGTTCGAAGGCATCTATAC | [50] |
|  |  | BxeB2031_HpaDRv | TGCTGATCCGCATTCATGTAG |  |
| *ftsZ* | Bxe_A0491 | FtsZFw | CGATTACGGTGCGCTGCATA | [50] |
|  |  | FtsZRv | ATGCCGGAATGTCGTACGTG |  |
| *gyrR* | Bxe_A4460 | GyrFw | GGGCAAGGACGAACGGTATT | [50] |
|  |  | GyrRv | ACAGATAAGCCCGAGCCAAC |  |

| **Additional file 1: Table S2. Protein identification and quantification data from the mass spectrometry analysis in *P. xenovorans.***  **Please visit https://zenodo.org/records/10814965**  **Additional file 1: Table S3. Proteins detected during growth on 3-HPA and 4-HPA but not in the control condition (“on”)** | | | | |  |
| --- | --- | --- | --- | --- | --- |
| **Locus Tag** | **Function** | **p2-*fldX1*** | | **WT-p2** | |
|  |  | **3-HPA / glucose** | **4-HPA / glucose** | **3-HPA / glucose** | **4-HPA / glucose** |
| **Aromatic compounds metabolism** | |  |  |  |  |
| Bxe_A2723 | Maleylacetoacetate isomerase HmgC | on | on | on | N.i. |
| Bxe_A0294 | 1,2-phenylacetyl-CoA epoxidase subunit H | on | N.i. | on | N.i. |
| Bxe_A0292 | 1,2-phenylacetyl-CoA epoxidase subunit I | on | N.i. | on | N.i. |
| Bxe_A0293 | 1,2-phenylacetyl-CoA epoxidase subunit J | on | on | N.i. | N.i. |
| Bxe_B1666 | FAD-dependent oxidoreductase (belongs to the GcvT family) | on | N.i. | N.i. | N.i. |
| Bxe_A0432 | Extradiol ring-cleavage dioxygenase class III protein subunit B | on | on | on | on |
| Bxe_B0884 | Shikimate 5-dehydrogenase | N.i. | on | N.i. | on |
| Bxe_A2169 | TauD/TfdA family dioxygenase (PFAM Taurine catabolism dioxygenase TauD TfdA) | on | on | on | on |
| Bxe_B0440 | Putative lactoylglutathione lyase (glyoxalase/Bleomycin resistance protein/Dioxygenase superfamily) | on | on | on | on |
| Bxe_C0213 | FAD-dependent monooxygenase (Salicylate hydroxylase) | N.i. | on | on | on |
| **Energy production & biosynthesis** | |  |  |  |  |
| Bxe_B0441 | Ribulose-bisphosphate carboxylase large subunit family protein | on | on | on | on |
| Bxe_B0431 | 4'-phosphopantetheinyl transferase superfamily protein | N.i. | on | N.i. | on |
| Bxe_B1637 | Ubiquinol oxidase subunit II | on | on | on | on |
| Bxe_A1420 | 3,4-dehydroadipyl-CoA semialdehyde dehydrogenase | on | off | off | off |
| Bxe_A2493 | FMNH2-dependent alkanesulfonate monooxygenase (catalyzes the desulfonation of aliphatic sulfonates) | on | on | on | on |
| Bxe_B2718 | Aldehyde dehydrogenase | on | on | on | on |
| **Transport proteins** | |  |  |  |  |
| Bxe_A2975 | Hypothetical protein DR64_656. Sugar ABC transporter (polysaccharide deacetylase) | on | N.i. | on | N.i. |
| Bxe_B0438 | Tripartite ATP-independent periplasmic transporter solute receptor, DctP family | on | on | on | on |
| Bxe_B0620 | OmpC family outer membrane porin (PFAM porin Gram-negative type) | on | on | on | on |
| Bxe_B0430 | Major facilitator superfamily MFS_1 | N.i. | on | N.i. | on |
| Bxe_A3901 | MFS transporter | on | on | on | on |
| **Transcriptional regulators** | |  |  |  |  |
| Bxe_A2960 | Heavy metal response regulator transcription factor IrlR | on | on | on | on |
| **Cellular Processes, Motility and Signaling** | |  |  |  |  |
| Bxe_B2971 | Fimbria/pilus periplasmic chaperone (pili assembly chaperone PapD, C-terminal domain) | on | on | on | on |
| Bxe_B1630 | YXWGXW repeat-containing protein. YXWGXW repeat (2 copies) | on | on | on | on |
| Bxe_B2969 | Type 1 fimbrial protein | on | on | on | on |
| Bxe_B2972 | Type-1 fimbrial protein | on | on | on | on |
| Bxe_A3608 | Anti-ECFsigma factor, ChrR. Cupin domain-containing protein. ChrR Cupin-like domain | N.i. | on | N.i. | on |
| **Other** |  |  |  |  |  |
| Bxe_A3609 | Hypothetical protein | N.i. | on | on | on |
| Bxe_B1225 | Hypothetical protein | on | N.i. | on | N.i. |
| Bxe_B0429 | Hypothetical protein. Protein of unknown function (DUF2783) | N.i. | on | N.i. | on |
| Bxe_B1587 | Hypothetical protein. Tetratricopeptide repeat | on | on | on | on |
| Bxe_B1588 | Hypothetical protein | N.i. | on | on | on |
| **No. of proteins induced** | | **24** | **26** | **24** | **24** |
| N.i., Not induced |  |  |  |  |  |

| **Additional file 1: Table S4. Proteins detected in the control condition (glucose) but not during growth on 3-HPA and 4-HPA (“off”)** | | | | | |
| --- | --- | --- | --- | --- | --- |
| **Locus Tag** | **Function** | **p2-*fldX1*** | | **WT-p2** | |
|  |  | **3-HPA / glucose** | **4-HPA / glucose** | **3-HPA / glucose** | **4-HPA / glucose** |
| **Oxidative stress response** | |  |  |  |  |
| Bxe_B2940 | Glutathione S-transferase | off | off | off | off |
| **Energy production & biosynthesis** | |  |  |  |  |
| Bxe_A2245 | UDP-glucose/GDP-mannose dehydrogenase family protein | off | off | off | off |
| Bxe_A1420 | 3,4-dehydroadipyl-CoA semialdehyde dehydrogenase. Aldehyde dehydrogenase | on | off | off | off |
| Bxe_A0730 | Mannitol dehydrogenase family protein (mannitol dehydrogenase rossman) | off | off | off | off |
| Bxe_A4273 | PFAM MaoC domain protein dehydratase | N.i. | N.i. | off | N.i. |
| Bxe_A3784 | PFAM UbiA prenyltransferase | N.i. | N.i. | N.i. | off |
| **Transport proteins** | |  |  |  |  |
| Bxe_B2964 | Sugar ABC transporter substrate-binding protein (eriplasmic binding proteins and sugar binding domain of LacI family) | off | off | N.i. | N.i. |
| Bxe_A3450 | PFAM binding-protein-dependent transport systems inner membrane component | off | off | off | off |
| Bxe_A3449 | PFAM binding-protein-dependent transport systems inner membrane component | off | off | off | off |
| Bxe_A3321 | Sodium:solute symporter family protein. Belongs to the sodium solute symporter (SSF) (TC 2.A.21) family | N.i. | off | off | off |
| Bxe_A0591 | GntP family permease (gluconate) | off | off | off | off |
| Bxe_A1248 | ABC transporter, transmembrane region | off | off | off | off |
| Bxe_A2316 | Channel that opens in response to stretch forces in the membrane lipid bilayer (may participate in the regulation of osmotic pressure changes within the cell) | off | off | off | off |
| **Transcriptional regulators** | |  |  |  |  |
| Bxe_A0967 | Regulatory protein LysR | N.i. | N.i. | off | N.i. |
| Bxe_B2375 | LysR family transcriptional regulator | off | off | off | N.i. |
| Bxe_A3026 | LysR family transcriptional regulator | N.i. | off | off | off |
| Bxe_A0659 | MarR family transcriptional regulator | off | N.i. | off | N.i. |
| **Cellular Processes, Motility and Signaling** | |  |  |  |  |
| Bxe_B1174 | Peptidase S10 (serine carboxypeptidase) | off | off | off | off |
| Bxe_A1540 | Oxygen sensor histidine kinase FixL (PFAM ATP-binding region, ATPase domain protein) | off | off | off | off |
| Bxe_A1851 | PAS domain-containing sensor histidine kinase (histidine kinase, dimerisation and phosphoacceptor region) | off | off | off | off |
| Bxe_A0162 | Flagellar export protein FliJ | off | off | off | off |
| Bxe_A0151 | Flagellar hook-associated protein FlgL | N.i. | off | N.i. | off |
| **Transcription and Repair processess** | |  |  |  |  |
| Bxe_B0234 | RNA polymerase sigma factor, sigma-70 family | off | off | off | off |
| Bxe_A0106 | DNA-3-methyladenine glycosylase I (Pfam Methyladenine glycosylase) | off | off | off | off |
| Bxe_C0187 | Ribbon-helix-helix domain-containing protein (CopG family) | off | off | N.i. | off |
| Bxe_B0806 | zf-TFIIB domain-containing protein (transcription factor zinc-finger) | off | off | off | N.i. |
| **Other** |  |  |  |  |  |
| Bxe_A2831 | Hypothetical protein | off | off | off | off |
| Bxe_A0268 | Hypothetical protein | off | off | off | off |
| Bxe_A2258 | Hypothetical protein | N.i. | off | N.i. | N.i. |
| **No. of proteins induced** | | 21 | 25 | 24 | 22 |
| N.i., Not induced |  |  |  |  |  |
